# Triterpenes Drug Delivery Systems, a Modern Approach for Arthritis Targeted Therapy

**DOI:** 10.3390/ph17010054

**Published:** 2023-12-28

**Authors:** Célia Faustino, Noélia Duarte, Lídia Pinheiro

**Affiliations:** Research Institute for Medicines (iMed.ULisboa), Faculdade de Farmácia, Universidade de Lisboa, Avenida Prof. Gama Pinto, 1649-003 Lisbon, Portugal; cfaustino@ff.ulisboa.pt

**Keywords:** triterpenes, drug delivery systems, arthritis, osteoarthritis, gout

## Abstract

Arthritis is a major cause of disability. Currently available anti-arthritic drugs, such as disease-modifying anti-rheumatic drugs (DMARDs), have serious side-effects associated with long-term use. Triterpenoids are natural products with known anti-inflammatory properties, and many have revealed efficiency against arthritis both in vitro and in vivo in several animal models, with negligible cytotoxicity. However, poor bioavailability due to low water solubility and extensive metabolism upon oral administration hinder the therapeutic use of anti-arthritic triterpenoids. Therefore, drug delivery systems (DDSs) able to improve the pharmacokinetic profile of triterpenoids and achieve sustained drug release are useful alternatives for targeted delivery in arthritis treatment. Several DDSs have been described in the literature for triterpenoid delivery, including microparticulate and nanoparticulate DDSs, such as polymeric micro and nanoparticles (NPs), polymeric micelles, liposomes, micro and nanoemulsions, and hydrogels. These systems have shown superior therapeutic effects in arthritis compared to the free drugs and are similar to currently available anti-arthritic drugs without significant side-effects. This review focuses on nanocarriers for triterpenoid delivery in arthritis therapy, including osteoarthritis (OA), rheumatoid arthritis (RA) and gout that appeared in the literature in the last ten years.

## 1. Introduction

Arthritis is an acute or chronic joint condition usually marked by stiffness, pain, swelling and restricted movement [1,2]. With over 100 different types, osteoarthritis (OA) stands as the most prevalent, particularly among older adults, causing non-inflammatory degeneration and significant disability. With aging, the incidence of OA rises, posing substantial challenges to health, society, and the economy [1,3]. Inflammatory arthritis can be caused by several factors, such as autoimmune processes (e.g., rheumatoid arthritis (RA), crystal deposition-induced inflammation (e.g., gout), infections (e.g., septic arthritis), other autoimmune connective tissue diseases (e.g., systemic lupus erythematosus) and extra-articular comorbidities [1,2]. The aetiology of arthritis depends on its specific type and is shaped by a complex interplay of genetic, epigenetic, and environmental factors. [2,3]. A patient’s quality of life can be negatively impacted by severe arthritis, which can also cause chronic pain and disability, interfere with everyday activities, and affect work performance [1]. Data from the recent Global Burden of Disease Study 2019 [4] showed a substantial increase in arthritis prevalence, incidence and years lived with disability (YLDs) from 2010 to 2019 (Table S1, Supplementary Materials) among the musculoskeletal diseases.

Triterpenoids are natural compounds with well-known anti-inflammatory characteristics. Several of them have shown in vitro and in vivo effectiveness against arthritis in a number of in vitro and in vivo models, with minimal cytotoxicity [5,6,7]. Anti-arthritic triterpenoids can be used therapeutically but their limited bioavailability, due to low water solubility and substantial metabolism after oral administration, are considerable drawbacks [6,8,9]. Therefore, drug delivery systems (DDSs) able to improve the pharmacokinetic profile of triterpenoids and achieve sustained drug release are useful alternatives for targeted delivery in arthritis treatment.

This review will focus on nanocarriers for the delivery of triterpenoids in the treatment of arthritis, including osteoarthritis (OA), rheumatoid arthritis (RA), and gout that have been reported in the literature in the last ten years. For a deeper understanding of the topic some aspects of these disorders will also be covered, as well as the various animal models used in the preclinical research studies.

The literature search was carried out from June to August 2023 on Web of Science, ScienceDirect, and PubMed, using several combinations of keywords and truncation (e.g., combinations of tritepenes, drug delivery systems, arthritis, inflammation, bioavailability, and pharmacokinetics). The timespan from 2013 to 2023 was considered. No restrictions on language or the geographical origin of authors were applied, and only peer-reviewed research articles or reviews were considered. Mendeley Reference Manager Software (version 1.19.8, 2020) was used to manage the references and eliminate duplicates.

### Types of Arthritis

**Figure 1:**
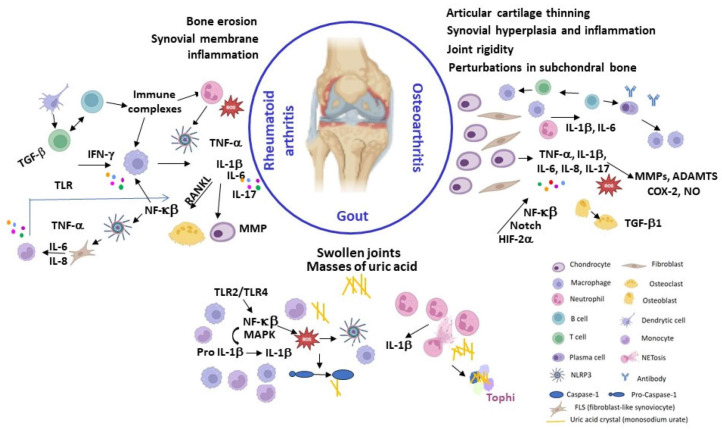
Summary of some clinical and pathogenic features of RA, AO, and Gout. Created with BioRender.com (accessed on 16 December 2023).

There are over a hundred varieties of arthritis, with osteoarthritis, a non-inflammatory degenerative form of the disease, being the most common. Rheumatoid arthritis (RA) is the most prevalent autoimmune inflammatory form of arthritis. Other causes of inflammatory arthritis include infections (like septic arthritis) or inflammation brought on by crystal deposition (like gout or pseudogout). Other autoimmune connective tissue disorders, such as systemic lupus erythematosus, and extra-articular comorbidities have also been linked to inflammatory arthritis. Figure 1 summarizes some of the clinical and pathogenic features of RA, AO, and Gout, which will be described below.

Osteoarthritis (OA) has traditionally been classified as non-inflammatory arthritis and considered a “wear and tear” disease, which is mainly cartilage-driven and usually a slowly progressive chronic disease typical of middle-aged to elderly people. Nonetheless, OA can also affect younger individuals, especially those that have suffered joint and/or bone injury or are involved in physically demanding activities. Several common injuries have been associated with post-traumatic OA, mainly involving the knee, including anterior cruciate ligament ruptures, meniscus tears, and patellar dislocation [1]. OA is a complex multifactorial disease with a strong genetic component estimated to account for 40–70% of the risk of developing OA, depending on the phenotype [10]. Several genes implicated in OA susceptibility and progression have been identified by genome-wide association studies of single nucleotide polymorphisms, many of which are involved in the formation, homeostasis and maintenance of cartilage and bone [11,12,13].

OA can cause cartilage damage, bone remodelling, synovial hyperplasia, and chronic inflammation, leading to pain, stiffness, and mobility loss. Moreover, synovial inflammation (synovitis) has been shown to precede structural changes in early OA [14]. OA can affect any joint, but it is more frequent in the weight-bearing joints, such as the knees and hips, and in frequently used joints, like the hands and spine (Table S2, Supplementary Materials). Caucasians are more likely to develop hand OA than Afro-Americans [1], while a low prevalence of hip OA has been observed in some Asian populations [3].

Among lifestyle factors, obesity is a major contributor to OA onset and progression, due to both excessive joint loading and metabolic effects. OA has also been associated with increased rates of comorbidities (Table S2, Supplementary Materials), which often contribute to faster deterioration and to decreased quality of life [3,10].

OA onset and progression is driven by a combination of (epi)genetic and environmental risk factors, such as aging, mechanical overload, and obesity. Biomechanical, metabolic and/or oxidative stress can disrupt cartilage homeostasis leading to chondrocyte shift to a hypertrophic phenotype associated with aberrant production of pro-inflammatory cytokines (IL-1β, IL-6 and TNF-α) and proteolytic enzymes responsible for the degradation of the collagen- and proteoglycan-rich extracellular matrix (ECM), fibrillation and erosion of the articular surface, apoptosis, matrix calcification, osteophyte formation and vascular endothelial growth factor (VEGF)-mediated angiogenesis and vascular invasion [14,15]. There is currently no cure for OA, and available treatments aim to reduce pain and improve function of the affected joints, which usually requires a combination of physical measures and pharmacotherapy, with late replacement surgery as last resource [2,3,16]. There is also an urgent need for truly disease-modifying osteoarthritis drugs able to delay or reverse disease progression. A summary of the pharmacotherapy together with other complementary treatments and lifestyle recommendations is described in (Table S2, Supplementary Materials) [2,3,17,18,19,20].

Rheumatoid arthritis (RA) is an autoimmune disorder characterized by chronic and systemic inflammatory processes. The prevalence of rheumatoid arthritis (RA) increases with age, and women have a 2 to 3-fold higher likelihood of developing RA compared to men (Table S1, Supplementary Materials). It predominantly impacts the articulations, giving rise to synovial inflammation and consequent deterioration of articular structures, which leads to discomfort, articular pain, and disability that could lead to deformities and loss of function if not treated [2,21]. RA typically manifests as a symmetrical affection of peripheral joints, particularly those of the hands, wrists, and feet, but it may progress to involve proximal joints [2,21]. The inflammatory response associated with RA is marked by an acute-phase reaction, denoted by increase of serum concentrations of C-reactive protein (CRP) and an elevated erythrocyte sedimentation rate (ESR), which are biomarkers for the assessment of the disease’s status. Furthermore, RA is characterized by a state of systemic inflammation that may cause several extra-articular comorbidities described in Table S2 (Supplementary Materials) [2,22].

The aetiology and pathogenesis of RA are very complex but it seems that the onset and progression of the disease are probably the consequence of the interaction between (epi)genetics [23,24,25], the existence of autoantibodies [26], and environmental factors, such as smoking, fine particulate air pollutants exposure, and periodontal disease [2,21,27]. An exhaustive description of pathogenesis is beyond the scope of this review but valuable information can be found in recent reviews [24,25,28,29,30]. Nowadays, RA can be effectively managed with a combination of medication and a healthy lifestyle (Table S2, Supplementary Materials) [27]. Early diagnosis and treatment are essential to achieve remission or low disease activity. Initial treatment involves the use of disease-modifying anti-rheumatic drugs (DMARDs) able to delay or even halt disease progression, preventing radiographic progression and improving function and quality of life [21,31]. These are often used in combination with NSAIDs or low-dose glucocorticoids (e.g., prednisone, prednisolone, dexamethasone, betamethasone, and triamcinolone) to reduce pain and inflammation while the disease remains active [21,31].

Gouty arthritis (gout) is an autoinflammatory disease caused by the deposition of needle-shaped monosodium urate (MSU) crystals in articular and non-articular structures [2,32,33]. The innate immune response to the urate crystal deposits triggers severely painful and self-debilitating acute gout flares, typically self-limiting within 7–14 days, alternating with asymptomatic intercritical periods [2,33]. Gout flares are usually monoarticular and typically affect a lower limb joint, frequently the big toe. Advanced disease is characterized by subcutaneous nodules (tophi) mainly composed of MSU, chronic articular inflammation, structural joint damage, and impaired mobility [2,33]. Gout resulted in 1.67 million global YLDs in 2019 [4] (Table S1, Supplementary Materials), with a higher gout prevalence being found in Han Chinese, New Zealand Maori and the Polynesian population [34]. Men are nearly three times more likely to develop gout than women (Table S1, Supplementary Materials) [4]. Hyperuricaemia (serum urate concentrations > 7 mg/dL) is the most important risk factor for gout development (Table S2, Supplementary Materials) [2,33]. Other risk factors for gout include advancing age, male sex, and ethnicity (Table S2, Supplementary Materials) [33,34]. The excess urate can also deposit in other tissues or organs, such as the kidneys, forming kidney stones (nephrolithiasis) [33,35]. A purine-rich diet (e.g., red meat and shellfish), alcohol intake (especially purine-rich beer), and consumption of high-fructose sweeteners result in increased urate production and can trigger a gout flare [33,35]. Gouty arthritis can be effectively controlled with proper medication, self-management strategies, and modification of lifestyle factors. Treatment of gout flares mainly involves early administration of anti-inflammatory drugs (e.g., NSAIDs) to relieve pain, suppress joint inflammation, and restore function (Table S2, Supplementary Materials) [33,36,37]. Gout flares refractory to conventional anti-inflammatory therapy may be treated with adrenocorticotropic hormone [37] or IL-1 inhibitors, initially developed as biologic DMARDs [38], such as canakinumab and anakinra, the latter with an improved cardiovascular safety profile [33,36]. Lowering serum urate levels typically employs anti-hyperuricemic agents that either reduce urate production or promote its excretion. XOD inhibitors (e.g., allopurinol and febuxostat) are first-line ULT agents that hinder MSU production [36,39]. Uricosuric agents (probenecid, benzbromarone, lesinurad, sulfinpyrazone and dotinurad), which promote renal urate excretion, are used as adjunctive ULT for patients with inadequate response to XOD inhibitor monotherapy or as second-line agents when XOD inhibitors are not tolerated [36]. Recombinant uricases (e.g., pegloticase), which metabolize urate to the more water soluble allantoin, are third-line ULT agents reserved for treatment of severe gout refractory to oral ULT [36,40].

## 2. Animal Models of Arthritis

Animal models play a significant role in preclinical studies concerning drug bioavailability, pharmacokinetic profile, efficacy, and toxicity. However, large interspecies difference in the expression of metabolic enzymes and drug elimination pathways, which influence both drug pharmacokinetics and pharmacology, requires careful selection of the animal model. Several animal models of arthritis, either experimentally induced or spontaneous, have been developed that significantly contributed to understanding disease pathogenesis and permitted evaluation of new potential anti-arthritic agents, despite no single animal model being able to fully replicate all the clinical features of human arthritis [7,41,42,43,44,45]. Both spontaneous and induced animal models of arthritis are available. The most frequently used animal models of induced arthritis are summarized in Table S1 (Supplementary Materials). Naturally occurring arthritis has been reported in aged mice, guinea pigs, rabbits, pigs, dogs, sheep, goat, horses, and macaques. Although naturally occurring arthritis models more closely mimic the onset and progression of human arthritis, they are slowly progressive, require animal skeletal maturity, and can be costly and time-consuming [41,42].

Spontaneous arthritis can also be modelled using genetically engineered animals, including transgenic and gene knockout/knockin mice. Transgenic mice over-expressing human TNF-α spontaneously develop chronic inflammatory erosive polyarthritis 4–6 weeks after birth [41,43,45,46]. SKG transgenic mice develop inflammatory polyarthritis at 2 months of age (or 2 weeks after zymosan injection), due to a point mutation in the ZAP-70 gene involved in T cell receptor signalling, with production of R_F_ and autoantibodies against CII [41,43,45,46]. More recently, the K/BxN spontaneous mouse model of arthritis (RA) has been developed by crossing T cell receptor (TCR) transgenic KRN mice with non-obese diabetic (NOD) mice expressing the MHC class II molecule Ag^7^. K/BxN mice develop severe and destructive inflammatory polyarthritis after the age of 4 weeks with high titers of autoantibodies against glucose-6-phosphate isomerase, and serum transfer to naïve mice induces arthritis in 7–14 days [41,43,45,46].

Genetically modified models of arthritis are highly reproducible and represent valuable tools to identify the biological mechanisms, molecular pathways, and potential targets involved in the disease pathogenesis, but are not as suitable for the evaluation of therapeutic agents since a single gene mutation does not account for the complex interplay between several signalling pathways that contribute to human arthritis. Therefore, induced models of arthritis are preferable for the assessment of anti-arthritic agents. These models involve mechanically induced joint destabilization or chemical treatment, usually by intra-articular injection of the chemical agent, causing an inflammatory reaction that mimics arthritis development [7,41,42,44,45]. The knee is the joint most often employed in animal models of arthritis [41,42].

Models of induced arthritis predominantly employ small animals, mainly mice, rats, and guinea pigs. Mechanical destabilization of the joint is usually surgically induced (e.g., anterior cruciate ligament transection, medial meniscus tear, partial meniscectomy), mimicking post-traumatic osteoarthritis [41,42]. Non-invasive techniques, such as tibial overload compression, intra-articular tibial plateau fracture, cyclic articular cartilage tibial compression and trans-articular impact, require expensive machinery and are less often used [42]. Chemically induced models are less invasive than those surgically induced and can be performed in a shorter timeframe, however, their pathophysiology does not correlate with the chronic and slowly progressive degeneration in human arthritis. These models can be valuable tools in RA research due to the inflammatory reaction they evoke and the induced immune response [41,43,45,46].

Monoiodoacetate (MIA) is often used to induce knee osteoarthritis upon intra-articular injection, mimicking human joint pathology and osteoarthritic pain [41,42]. On the other hand, injection of MSU crystals (suspended in saline or in mineral oil) is the commonly employed method to trigger acute gout inflammation and gouty arthritis [7,44]. Immunization with immunogenic adjuvants, such as complete Freund’s adjuvant (CFA) and cartilage antigens (e.g., CII) by intradermal or subcutaneous injection, induces immune-mediated chronic polyarthritis of systemic nature that resembles RA [41,43,45,46].

## 3. Triterpenoids with Activity on Osteoarthritis, Rheumatoid Arthritis and Gout

In a recent review, we gathered information on several triterpenic compounds as promising agents for the treatment of rheumatoid arthritis [5]. Celastrol, betulinic acid, nimbolide, and several ginsenosides stand out as potential drug candidates. Animal studies have demonstrated that their bioactivity resulted from a reduction in several immunological, hematological, and biochemical markers, and RA activity indices, namely, paw edema, arthritis scores, and body weight. In this section, we further expand the information on antiarthritic triterpenes, beginning with an exploration of their bioactivity against osteoarthritis and gout.

**Figure 2:**
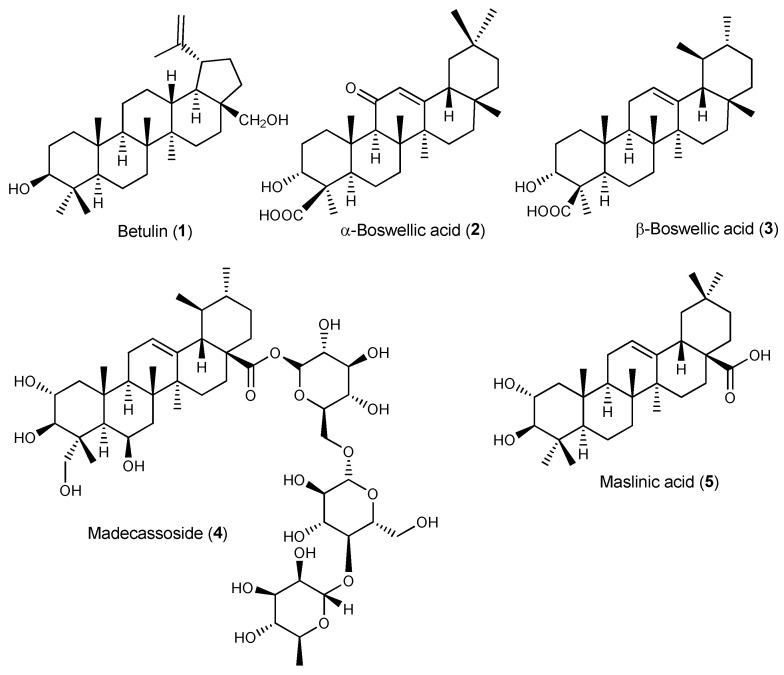
Structures of triterpenes with activity on osteoarthritis and gout.

Betulin (**1**, Figure 2) has been used in traditional medicine for inflammation treatment [47,48]. Betulin inhibited IL-1β-induced gene expression, secretion and proteolytic activity of MMP-3, MMP-1 and MMP-13 in primary cultured rabbit articular chondrocytes and in vivo production of MMP-3 in the knee joint of rats upon intraarticular administration in the knee joint, thus protecting articular cartilage tissue (Table 1) [49]. Furthermore, **1** inhibited the expression of pro-inflammatory mediators (IL-6, TNF-α, PGE2 and NO) and the generation of COX-2 and iNOS induced by IL-1β in mouse chondrocytes in vitro [50]. It also down-regulated MMP-13 and ADMATS-5 expression while up-regulating expression of Collagen II and aggrecan, thus preventing the degradation of the extracellular matrix [50]. Mechanistic studies showed that **1** activated the AKT/Nrf2 pathway and hindered p65 phosphorylation. In vivo, **1** ameliorated osteoarthritis symptoms in IL-1β-induced rats by inhibiting cartilage destruction and inflammatory progression via the AKT/Nrf2/HO-1/NF-κB signal axis [50]. Betulin (**1**) has also been shown to suppress TNF-α and IL-1β production in synovial fibroblasts from OA patients undergoing total knee replacement surgery via inhibition of the MEK/ERK/NF-κB signalling pathway [51].

Boswellic acids are major constituents of *Boswellia serrata* gum resin extract known for their anti-inflammatory and immunomodulatory properties with potential anti-arthritic effects [48,52]. Increased lysosomal activity, lipid peroxidation, TNF-α levels, and paw volume in the MSU-induced inflammation mouse model were significantly reduced after treatment with commercially available boswellic acid (a mixture of the α- and β-boswellic acids, **2** and **3**, respectively) [53]. In vitro, boswellic acid decreased the level of β-glucuronidase and lactate dehydrogenase in MSU-stimulated human polymorphonuclear leucocytes (PMNL) (Table 1) [53].

Madecassoside (**4**), a triterpenoid saponin present in *Centella asiatica*, is a potent anti-rheumatoid agent despite its low bioavailability. Wang et al. have shown that **4** was able to enhance the secretion of anti-inflammatory cytokine IL-10 in the small intestine of CIA rats following oral administration, presumably by increasing the number of Foxp3(+) T lymphocytes in the lamina propria, thus ameliorating arthritis symptoms (Table 1) [54]. The authors proposed that intestinal IL-10 mobilization may represent a relevant mechanism contributing to the anti-arthritic effects of bioactive triterpenoids with poor oral bioavailability, such as madecassoside [54]. In gouty arthritis mouse models, **4** reduced MSU-induced paw swelling and joint inflammation in the footpad gout model as well as neutrophil infiltration, pro-inflammatory cytokine secretion, and NLRP3 expression in MSU-triggered peritonitis [20,55]. Moreover, **4** down-regulated serum uric acid, blood urea nitrogen and creatinine in hyperuricemic mice, improving renal dysfunction [55].

Olives and olive oil are rich in bioactive pentacyclic triterpenoids, mainly maslinic acid (**5**). In a randomized, double-blind, placebo-controlled trial, maslinic acid supplementation (50 mg daily for 12 weeks) has been shown to alleviate mild knee joint pain in the elderly [56], suggesting that olive products containing compound **5** can be useful preventive and therapeutic food ingredients for arthritis and other chronic inflammatory diseases. In vitro, **5** suppressed TNF-α production and gene expression levels of IL-1β, IL-6, iNOS and COX-2 in LPS-stimulated macrophages (RAW 264.7 cells) by inhibiting NF-κB activation. In the CAIA mouse model, **5** was able to ameliorate arthritis symptoms by down-regulating the mRNA expressions of pro-inflammatory cytokines in the synovial membrane of the mouse knee joints [57]. Further mechanistic studies showed reduced the expression of inflammatory cytokines in the synovium through inactivation of TLR and NF-κB signalling pathways and down-regulation of leukotrienes via the glucocorticoid receptor (Table 1) [58].

**Table 1:** Natural triterpenoids with activity on osteoarthritis and gout.

Triterpenoid	Cell Model/Animal Model/Dosage	Effects and Mode of Action	Ref.
Betulin (**1**)	IL-1β-stimulated OA mouse primary chondrocytes; pretreated with betulin (0, 25, 50 and 100 μM) for 2 h then stimulated with IL-1β (10 ng/mL) for 24 h DMM in C57BL/6 male wildtype mice (n = 6); after DMM surgery, mice were administered betulin (20 mg/kg/day, i.p.) or inert carrier (DMSO) for 8 weeks.	Inhibition of the activation of NF-κB signal in OA mouse chondrocytes, reduction of the expression of iNOS and COX-2 and inhibition of the generation of NO, PGE2, TNF-α and IL-6. Reversion of the expression of collagen II and reduced protein levels of MMP13 and ADAMTS5 in a dose-dependent manner, preventing ECM degradation. Promotion of AKT phosphorylation, enhanced nuclear expression of Nrf2 and up-regulated HO-1 expression. Prevention of the progression of DMM-induced OA in mice.	[50]
	Human synovial fibroblasts from OA patients treated with betulin (0, 1, 3 and 10 μM) for 24 h.	Inhibition of TNF-α and IL-1β expression in human OA synovial fibroblasts via MEK/ERK/NF-κB signalling	[51]
	Primary cultured rabbit articular chondrocytes (from male New Zealand white rabbits); pretreated with betulin (0, 1, 10, 50 or 100 μM) for 2 h before incubation with IL-1β (10 ng/mL) for 24 h Male SD rats; pretreated with intraarticular injection (30 μL) of betulin (50 or 100 μM) in the right knee joint 3 h before intraarticular injection (30 μL) of IL-1β (20 ng)	Inhibition of IL-1β-induced gene expression, secretion and proteolytic activity of MMP-3, MMP-1, MMP-13, ADAMTS-4 and ADAMTS-5 while increasing gene expression of type II collagen in primary cultured rabbit articular chondrocytes In vivo chondroprotective effects (intraarticularly administration) by suppressing IL-1β stimulated MMP-3 production in articular cartilage tissue of the rat knee joint.	[49]
Mixture of α- and β-Boswellic acid (**2** and **3**)	MSU-stimulated human polymorphonuclear leucocytes (PMNL); Cells pre-incubated with boswellic acid (0, 50 or 100 μg/mL) or indomethacin (10 μg/mL) for min before addition of MSU crystals (1 mg/mL) and further incubation for 30 min at 37 °C MSU crystal-induced inflammation in Swiss albino mice; intraperitoneal administration of boswellic acid (30 mg/kg) or indomethacin (3 mg/kg), 1 h before intradermal injection (0.2 mL) of MSU crystal suspension (4 mg) into the right footpad, daily, for 3 days.	In vitro decrease of the level of β-glucuronidase and lactate dehydrogenase in MSU-treated PMNL cells in a concentration-dependent manner. Significant in vivo reduction of the increased lysosomal activity, lipid peroxidation, TNF-α levels and paw volume in the MSU-induced inflammation mouse model.	[53]
Madecassoside (**4**)	Footpad inflammation or peritonitis in DBA/1 mice triggered by intradermal or intraperitoneal injection of MSU crystals, respectively. Potassium oxonate-induced hyperuricemia in ICR mice.	Inhibition of MSU-induced paw swelling and joint inflammation in the footpad gout model. Decrease of neutrophil infiltration, secretion of IL-1β, IL-6 and MCP-1, and NLRP3 expression in mice with MSU-induced peritonitis. Improvement of renal dysfunction and lowering of serum urate, blood urea nitrogen and creatinine levels in the hyperuricemic mouse model.	[55]
Maslinic acid (**5**)	CAIA in DBA/1J mice (n = 10); oral administration of maslinic acid (200 mg/kg/day) for 11 days, with CAIA induction on day 8 by i.p. injection with type II collagen monoclonal antibodies and booster LPS i.p. injection on day 11	Amelioration of arthritis symptoms by promoting tissue formation and reducing gene expression of inflammatory cytokines in the synovium via inactivation of TLR and NF-κB signalling pathways and down-regulation of leukotrienes (LTB4) through the glucocorticoid receptor.	[58]
	LPS-stimulated macrophages; treated with LPS (2 ng/mL) and maslinic acid (0, 10 or 20 μM) for 3 h CAIA in DBA/1J mice (n = 6); orally administered maslinic acid (100 mg/kg/day in corn oil) for 7 days before i.p. injection of 3 mg cocktail of 5 mAbs to type collagen followed by i.p. injection of LPS (50 μg) 3 days later	Suppression of TNF-α production and gene expression levels of IL-1β, IL-6, iNOS and COX-2 in LPS-stimulated RAW 264.7 cells by inhibiting NF-κB activation Significant reduction of the arthritic score of CAIA mice paw and inhibited the expressions of TNF-α, IL-1β and IFN-γ mRNA in the synovial membrane of knee joints	[56]

## 4. Triterpene Drug Delivery Systems for Arthritis Targeted Therapy

Several natural triterpenoids with anti-inflammatory and anti-arthritic activities described in recent literature [5,6,7] can be potential therapeutic alternatives and/or adjuvants for anti-arthritic drugs currently in the market, which have unwanted side-effects associated with prolonged use. However, natural products, including triterpenoids, often suffer from poor absorption and low bioavailability due to extensive first-pass metabolism upon oral administration, and low water solubility, among other physicochemical characteristics (Table S4, Supplementary Materials) [6,8,9]. Drug delivery systems (DDSs), particularly nanotechnology-based DDSs, represent a promising approach to improving natural triterpenoids’ bioavailability, efficacy, and pharmacokinetic profile. Moreover, nanocarriers can protect triterpenoid drug candidates against degradation, enhance water solubility, reduce drug toxicity, deliver the drug to its specific target(s), and achieve controlled and/or sustained drug release at the target site [8,9,59].

Nanoparticles (NPs) exhibit unique structural, physical, chemical, and biological properties due to their very small (nano) size and high specific surface area. Several techniques, including extrusion, sonication, homogenization and/or freeze-thawing can be used to produce NPs with well-defined sizes [8,59]. Composition, particle size, size distribution and surface charge are determinants for NP stability, uptake, biodistribution, clearance, cytotoxicity, drug loading, and drug release properties [8,59].

NPs usually show enhanced oral bioavailability attributed to enhanced luminal residence time due to their small size and high surface area, improved adhesion to the gut mucosa, and enterocyte uptake involving non-specific endocytosis pathways (e.g., clathrin-dependent endocytosis, caveolae-mediated endocytosis and micropinocytosis). Positively charged NPs show enhanced uptake by intracellular pathways compared to neutral and negatively charged NPs. Furthermore, NPs with sizes around 100–200 nm show an enhanced permeability and retention (EPR) effect that has been used for passive targeting of tumours based on their leaky vasculature and poor lymphatic drainage [8,9,59]. Similarly, persistent inflammation in arthritis increases vascular permeability, leading to selective accumulation of nanocarriers in the inflamed synovial tissue via the extravasation through leaky vasculature and the inflammatory cell-mediated sequestration (ELVIS) effect [60].

Serum protein adsorption on the surface of NPs promotes opsonization and sequestration by the mononuclear phagocyte system (MPS), leading to rapid clearance from the bloodstream and accumulation in the liver and spleen. The rate of cell uptake is higher for positively charged NPs compared with neutral and negatively charged ones [8,9,59]. NPs below 100 nm can escape clearance by the MPS and tissue-resident phagocytic cells, while NPs with a hydrodynamic diameter below 6 nm, corresponding to the kidney filtration threshold, undergo fast renal clearance [8,59]. NPs with positive surface charges are more rapidly cleared via the renal route compared to negatively charged NPs [8,59]. Surface functionalization with hydrophilic polymers, namely PEG (PEGylation), confers steric stabilization and prevents protein opsonization, providing stealth NPs with prolonged blood circulation time and a high rate of extravasation into permeable tissues [8,9,59,60,61]. Another strategy to improve blood residence time involves NP coating with endogenous biomaterials, such as albumin or red blood cell membrane, producing biomimetic NPs which are recognized as “self” by macrophages in MPS [60,61]. Moreover, cell membrane-coating can endow NPs with specific functions of the original cell, including targeting ability with subsequent drug delivery selectively to the target site(s), such as the inflamed synovium in arthritis [60,61].

Additionally, cell surface receptors, lipid components of the cell membrane, and proteins or antigens on cell surfaces provide targets for active drug delivery, reducing nonspecific distribution and undesired side-effects. Active targeting can be achieved by NP functionalization with peptides, proteins, antibodies, aptamers or other appropriate ligands for enhanced or specific cellular uptake [8,9,59,62,63]. Peptide ligands, such as the RGD peptide family, which bind the α_v_β_3_ integrin receptor over-expressed on neovascular endothelial cells, have been used to target angiogenesis in the inflamed joint synovium, which is typical of RA [60,61]. Active targeting of macrophages in RA has been accomplished by surface modification of NPs with ligands like hyaluronan [64], folate [65,66], and dextran sulphate [67], which bind CD44, folate receptor β, and scavenger receptors, respectively, overexpressed on the surface of activated macrophages [60,61]. Furthermore, stimuli-responsive nanocarriers can be developed to improve targeting and achieve triggered or controlled drug release by using endogenous (e.g., pH, redox, enzymes) or exogenous (e.g., temperature, light, ultrasound, magnetic fields) stimuli [8,9,59,64,66]. Other active targeting approaches include conjugation with cell-penetrating peptides and cell membrane-coated NPs [60,61,66]. Biomimetic NPs that result from coating with cell membrane of neutrophiles [68], macrophages [69], and regulatory fibroblast-like synoviocytes [70] are recent promising strategies to enhance NP inflammatory (joint) targeting ability in arthritis.

**Figure 3:**
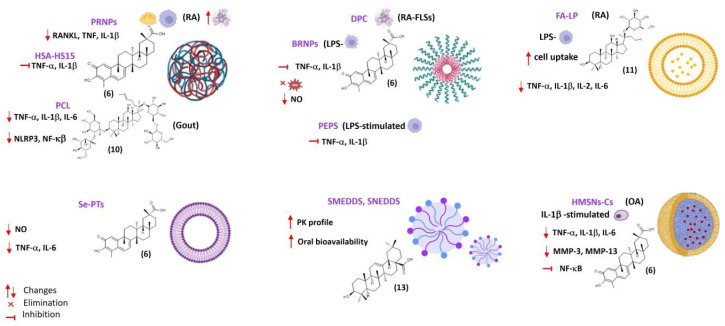
Some DDS for the main triterpenic compounds in rheumathoid arthritis, osteoarthritis, and gouty arthritis. Created with BioRender.com (accessed on 19 December 2023).

Among the different types of nanocarriers that have been developed for drug delivery, polymeric NPs, polymer-drug conjugates, micelles, liposomes, lipid NPs, and nanoemulsions are the most often used for the delivery of natural products [8,9,59]. Several natural products and natural product-derived drugs currently in the market are available as nanoformulations, including liposomal amphotericin B (antifungal), albumin-bound paclitaxel NPs (antitumoral) and pegloticase, a polymer-protein drug conjugate (PEGylated porcine-like uricase) used in the treatment of chronic gouty arthritis [8,71]. Several nanoparticulate DDSs comprising a wide variety of dosage forms, including polymeric nanoparticles, micelles, solid lipid nanoparticles, nanoliposomes, and self-emulsifying drug delivery systems have been developed for triterpenoid delivery in arthritis therapy, aiming to improve bioavailability and pharmacokinetic profile, and/or enhancing their anti-inflammatory and anti-arthritic effects. Table 2, Table 3, Table 4 and Table 5 and Figure 3 present collected nanoparticulate drug delivery systems involving antiarthritic natural triterpenoids (Figure 3).

### 4.1. Polymeric Nanoparticles

Polymeric nanoparticles include nanospheres and nanocapsules with sizes in the range 1–1000 nm that differ in their morphology [8,59]. Nanospheres consist of a (continuous) polymeric matrix in which the drug can be entrapped or surface-adsorbed while nanocapsules comprise a liquid core (water or oil), acting as a reservoir for the dissolved drug, and enclosed by a polymeric shell [8,59]. Biocompatible and biodegradable synthetic polymers have been used in the fabrication of polymeric NPs, such as PEG, polyvinyl alcohol (PVA), poly(caprolactone) (PCL), poly-L-lactic acid (PLA), and poly(D,L-lactic-co-glycolic acid) (PLGA), which are approved by the United States Food and Drug Administration (FDA) [8,59]. Bioresorbable PLGA is extensively used since it provides high drug loading (DL) and encapsulation efficiency (EE) and degrades into natural metabolites (lactic acid and glycolic acid) that are fully resorbed in the human body. Natural polymers, usually polysaccharides (e.g., chitosan, alginate, hyaluronan, cellulose and dextran) or proteins (e.g., albumin, collagen and gelatine) generally regarded as safe (GRAS) by the FDA, have also been used in alternative to synthetic polymers due to improved biocompatibility, biodegradability, and lower cytotoxicity [8,59]. Triterpenoid encapsulation into nanoparticles can improve water solubility, in vivo stability, oral bioavailability, efficacy, and safety profile [8,9,59]. Moreover, polymeric NPs can be functionalized with specific antibodies or with engineered antibody fragments that target macrophages in inflammatory arthritis, such as RA [8,59].

**Figure 4:**
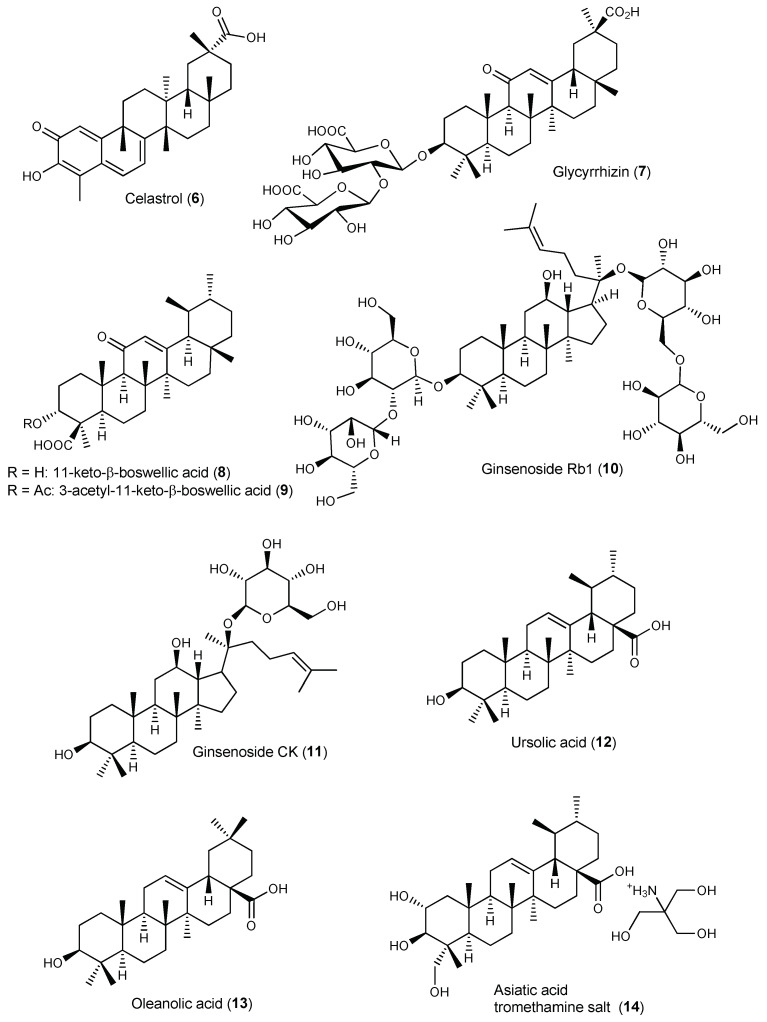
Structures of triterpenes incorporated in drug delivery systems (DDSs) with activity on arthritis.

Celastrol (**6**, Figure 4) is a promising anti-arthritic agent, despite its poor aqueous solubility and low bioavailability. Selective delivery of **6** to arthritic joints using enzyme-responsive nanoparticles (PRNPs) composed of RGD-modified PLGA nanoparticles (RNPs) covered with MMP-9-cleavable PEG chains has been attempted [72]. The RGD peptide (arginyl glycyl aspartate) is a ligand of the integrins, namely α_v_β_3_, which are adhesion glycoproteins serving as signalling receptors highly expressed on the surfaces of synoviocytes (fibroblasts, endothelial cells, and chondrocytes), synovial-infiltrating cells (macrophages, T cells, B cells, and neutrophils) and osteoclasts [72]. To avoid organ toxicity and selective uptake by endothelial cells, RNPs were modified with MMP-9-responsive PEG chains, and the resulting NPs (PRNPs) showed negligible off-target distribution [72]. In the presence of MMP-9, **6**-loaded PRNPs (**6**-PRNPs) showed high cellular uptake in both human synovial macrophages and osteoclasts derived from late-stage RA patients undergoing joint replacement surgery, via RGD-α_v_β_3_ integrin interaction, thus enhancing the apoptosis of the forementioned cells [72]. PRNPs have shown an arthritic joint-specific distribution and when loaded with celastrol (**6**) were able to reduce the number of osteoclasts and inflammatory macrophages in RA joints in the AA rat model. Moreover, inflammatory remission with bone erosion repair was observed in rats with advanced RA treated with **6**-PRNPs without significant side-effects [72].

Gong et al. developed human serum albumin (HSA)-Kolliphor^®^ HS15 nanoparticles (HAS-HS15 NPs) loaded with celastrol (**6**) (**6**-HSA-HS15 NPs) to overcome the limitations in targeted therapy for RA and improve the triterpenoid safety profile [73]. HSA as a nanocarrier is stable, biocompatible, biodegradable, and non-immunogenic, providing prolonged circulation in the bloodstream since it is not taken up by the MPS. Moreover, HSA may enhance drug absorption through the epithelium by facilitating diffusion, improving the drug pharmacokinetic profile and changing the drug tissue distribution pattern (Table S5, Supplementary Materials). Kolliphor^®^HS15 is a non-ionic surfactant with low toxicity used as pharmaceutical excipient due its solubilizing and emulsifying properties, which is suitable for parenteral and oral formulation of poorly water-soluble drugs. **6-**HSA-HS15 NPs accumulated at the inflammation site due to the ELVIS effect and the inflammatory targeting ability of albumin, which were subsequently phagocytosed by the activated macrophages at the inflamed joint of rats with adjuvant-induced arthritis [73]. This in situ release of celastrol (**6**) resulted in the inhibition of pro-inflammatory cytokines secretion. **6**-HSA-HS15 NPs exhibited superior therapeutic efficacy and significantly improved safety profile compared with free **6** and **6**-HSA NPs formulated without Kolliphor^®^HS15 [73].

Ansari et al. developed aminocellulose (AC)-grafted-polycaprolactone (PCL) coated gelatine NPs (PCL-AC-gel NPs) for co-delivery of glycyrrhizin (**7**, Figure 4) and budenoside against RA [74]. The inner core of the nanocarriers consisted of gelatine NPs loaded with hydrophilic glycyrrhizin (**7**) and the outer shell layer was composed of PCL-AC polymer encapsulating hydrophobic budenoside [74]. Drug release studies revealed a sustained release of both 7 and budenoside from dual-loaded NPs, with almost 50% of each drug being released within 24 h. The dual drug-loaded NPs reduced inflammatory cells infiltration, joint swelling, collagen destruction, and bone erosion in CIA rats [74]. The core-shell nanocarriers also decreased the levels of inflammatory mediators and exerted superior therapeutic efficacy compared to the free drugs, presumably due to the slow and sustained drug release and synergistic effects. Moreover, the NPs did not elicit toxic side-effects on liver and kidney in vivo [74].

Bairwa and Jachak developed PLGA NPs loaded with either 11-keto-β-boswellic acid (**8**) [75] or 3-O-acetyl-11-keto-β-boswellic acid (**9**) [76] to improve oral bioavailability and anti-inflammatory activity of the triterpenoids. In vitro release studies of both **8**-loaded and **9**-loaded NPs in PBS (pH 7.4) at 37 °C showed an initial burst release effect, which was attributed to surface association of the triterpenoids, followed by sustained release due to slow diffusion of the entrapped triterpenoid into the release medium [75,76]. Both **8**-loaded PLGA and **9**-loaded PLGA NPs NPs showed enhanced in vivo anti-inflammatory properties in carrageenan-induced hind paw oedema in rats (50 mg/kg p.o.) compared with free **8** and **9**, respectively. Oral bioavailability studies showed a 7-fold and 6-fold increase in bioavailability of **8** and **9**, respectively, when loaded in PLGA NPs compared with the corresponding free triterpenoid (Table S2, Supplementary Materials) [75,76].

Liu et al. encapsulated ginsenoside Rb1 (**10**) in polymeric nanocapsules to improve its oral bioavailability and enhance the therapeutic effect against gouty arthritis [77]. Under physiological conditions (PBS pH 7.40, 37 °C), nano-**10** showed a slow-release rate when compared with its free form. In rats with MSU-induced gouty arthritis, the nanoformulation inhibited the NLRP3 and NF-κB pathway, reduced the expression of pro-inflammatory cytokines in the inflamed rat joints, and ameliorated paw swelling and bone destruction more significantly than **10** alone [77].

### 4.2. Polymeric Micelles

Polymeric micelles are core-shell nanostructures made by self-assembly of amphiphilic block copolymers comprising a hydrophobic core and a hydrophilic shell. Loading of hydrophobic natural product drugs into the lipophilic core leads to enhanced drug stability and bioavailability. The hydrophilic shell, which provides water-solubility and intrinsic stealth effect, is usually made of PEG while the hydrophobic core is often made of poly(propylene oxide) (PPO), PLGA or PCL [8,9,59]. Polymeric micelles are thermodynamically stable colloidal systems with diameters in the range 10–100 nm, low polydispersity index (PDI), and critical micelle concentration (CMC) values between 10^−7^–10^−6^ mol L^−7^ [8,59]. For therapeutic purposes, chemical cross-linking of either shell or core domains is often necessary to decrease the CMC and obtain stable micelles, which avoids micellar dissociation with burst release of the encapsulated drug upon intravenous administration due to extensive blood dilution [8,59]. Targeted delivery can be achieved by designing stimuli-responsive polymeric micelles that deliver their therapeutic load at the target site upon changes in the environment, such as pH, temperature or light, or by surface functionalization with specific cell-targeting ligands [8,59].

Yu et al. developed dextran sulphate (DS)-based MMP-2 enzyme-sensitive nanomicelles targeting the activated macrophage scavenger receptor class A (SR-A) to improve celastrol (**6**) bioavailability and efficacy in RA treatment (Table 5) [78]. They designed an amphiphilic polymer prodrug named DPC using **6** as the hydrophobic core and DS as hydrophilic block as well as ligand of SR-A (also called CD204), which is mainly expressed on the surface of activated macrophages that are abundant in arthritic joints [78]. Celastrol (**6**) and DS were linked via the MMP-2-responsive peptide PVGLIG to achieve specific drug release through hydrolysis of the linker by MMP-2 enzyme overexpressed in inflamed joints of RA patients [78]. The DS-PVGLIG-**6** (DPC) self-assembled into nano-sized micelles were further loaded with **6**. The **6**-loaded nanomicelles (DPC@**6**) showed good targeting of activated macrophages and sustained-release effect in vitro when in the presence of MMP-2 enzyme. DPC@**6** had a higher inhibition rate of cell inflammatory factors and better therapeutic effects in AA rats compared with free **6** and negligible systemic toxicity [78].

Zhao et al. developed ROS-responsive polymeric micelles for **6** delivery by conjugating hydrophobic bilirubin (an endogenous antioxidant) to a hydrophilic PEG chain [79], taking advantage of excessive ROS production at inflamed sites in arthritic joints. The amphiphilic polymer self-assembled in aqueous solution at a CMC of 7 µg/mL to form nanoparticles (BRNPs) with a hydrodynamic diameter around 68.8 nm [79]. In vitro release studies showed faster and sustained-release of **6** from **6**-loaded BRNPs (**6**-BRNPs) in the presence of H_2_O_2_, with up to 60% **6** release over the first 4 h in 100 mM H_2_O_2_. **6**-BRNPs were able to scavenge intracellular ROS and down-regulated NO level in vitro after being effectively internalized by activated macrophages [79]. In vivo, **6**-BRNPs preferentially accumulated at inflamed joints of AA rats (presumably due to the long systemic circulation of PEGylated NPs and passive diffusion by extravasation through leaky vasculature and subsequent inflammatory cell-mediated sequestration), ameliorating joint swelling and bone erosion, and decreasing pro-inflammatory cytokine production [79]. Compared with free celastrol (**6**), **6**-BRNPs had enhanced anti-arthritic effect and lower systemic toxicity [79]. 

An et al. developed polymeric micelles for celastrol (**6**) delivery based on a reactive oxygen species (ROS) sensitive block copolymer poly(ethylene glycol)-block-poly(propylene sulfide) (PEG-b-PPS, denoted as PEPS) made of hydrophilic PEG and hydrophobic PPS [80]. PEG provides biocompatibility, stability, and prolonged circulation in the bloodstream while PPS, with free thiol group, makes the polymer ROS-responsive. In vitro oxidation-sensitive release of celastrol (**6**) from **6**-loaded PEPS following disassembly of the PEPS micelle was observed in PBS containing 0.3% *w*/*w* H_2_O_2_ for ROS generation [80]. Confocal microscopy showed that the NPs were readily internalized into LPS-activated macrophages (RAW264.7 cells) within 30 min in vitro while internalization in cells without LPS pre-activation was substantially less [80]. **6**-PEPS significantly decreased the production of pro-inflammatory cytokines in LPS-activated macrophages by inhibiting the NF-κB and Notch1 signalling pathways, thus avoiding macrophage polarization toward the pro-inflammatory M1 phenotype [80]. In vivo distribution studies in CIA mice showed that the polymeric micelles selectively accumulated in the inflamed limb joints upon intravenous administration [80]. Histopathological examination of joints showed marked reduction of synovial inflammation, bone erosion, and cartilage destruction when compared with untreated mice or mice treated with blank NPs [80]. Moreover, **6**-PEPS treatment decreased the levels of proinflammatory cytokines TNF-α, IL-1β and IL-6, and increased anti-inflammatory cytokines transforming growth factor beta 1 (TGF-β1) and macrophage colony-stimulating factor (M-CSF) levels in the serum of CIA mice without cytotoxic effects to the major organs (heart, liver, spleen, lung, and kidney) [80].

Goel et al. developed 3-acetyl-11-keto-β-boswellic acid (**9**)-loaded polymeric nanomicelles for topical anti-inflammatory and anti-arthritic activity using *N*-isopropylacrylamide, vinylpyrrolidone, and acrylic acid [81]. The nanoformulation allowed for sustained release of **9** at physiological pH of 7.4 and achieved a 3-fold increase in rat skin permeability in vitro compared with gel containing the same amount of plain phytochemical, showing enhanced in vivo anti-inflammatory and anti-arthritic activity in rats. The small size (45 nm) and mucoadhesiveness of the polymeric micelles also contributed to their superior therapeutic efficiency [81].

### 4.3. Vesicular Drug Delivery Systems

Vesicular drug delivery systems (VDDSs) are characterized by vesicle structure comprising one (or more) concentric or continuous bilayers that result from self-assembly of amphiphiles in aqueous media. VDDSs can incorporate both hydrophobic and hydrophilic drugs in the lipophilic bilayer or in the aqueous core, respectively, and are suitable for oral, parenteral, topical, and transdermal delivery. Vesicle composition, size, surface charge, and manufacturing process strongly influence the properties of VDDSs and impact their in vivo fate and effectiveness as drug carriers [82,83,84]. Vesicles are usually prepared by thin film hydration, reverse-phase evaporation, or high-pressure homogenization, using non-immunogenic, biocompatible, and biodegradable components, such as phospholipids and non-ionic surfactants, employed in the construction of liposomes and niosomes, respectively. Cholesterol is also present in the composition of most VDDSs for stabilization of the vesicle membrane, increasing the gel state phase transition temperature and providing increased rigidity, which reduces the permeability of the bilayer and thus avoids premature drug leakage [83,84]. VDDSs can enhance solubilization of poorly water-soluble bioactive phytochemicals, including triterpenes [83], provide sustained-release formulations, increase permeability across the epithelial membrane, and promote lymphatic transport, thus avoiding the hepatic first-pass effect, and improving oral bioavailability and the pharmacokinetic profile. Furthermore, encapsulation in VDDs provides protection against degradation, improving stability and reducing triterpenoid toxicity. Conventional VDDSs, such as liposomes and niosomes, have limited gastrointestinal stability, which can be improved by inclusion of bile salts into the bilayer constructs [82,83,84,85]. Gel formers (e.g., Carbopol, sodium alginate or Pluronic^®^ F127) can be added to produce vesicular gels suitable for topical and transdermal delivery [83].

#### 4.3.1. Liposomes

Liposomes are spherical vesicles comprising one (unilamellar) or multiple (multilamellar) concentric lipid bilayers surrounding an internal aqueous compartment, made of natural or synthetic phospholipids and cholesterol. Egg or soybean phosphatidylcholines (PCs) with long acyl chains (C16 or C18) are often used due to their structural resemblance with cell membrane phospholipids, providing biocompatibility, biodegradability, and a good safety profile. Liposomal systems are susceptible to fusion, aggregation, oxidation, and/or hydrolysis with subsequent drug leakage. PEGylation improves serum stability and produces stealth liposomes with prolonged circulation in the bloodstream. Coating with polysaccharides (e.g., chitosan, alginate, and pectin) improves mucoadhesion to intestinal epithelia, prolonging liposomal exposure in the small intestine, and thus promoting oral absorption [84].

Liposomal degradation in the gastrointestinal tract can be hindered by incorporation of bile salts (e.g., sodium deoxycholate, sodium glycocholate and sodium taurocholate) in the lipid bilayer, which lowers the phase transition temperature and enhances membrane flexibility and permeability [82,85]. The bile salt confers a negative charge to the vesicles, improving colloidal stability and promoting drug uptake through M-cells in the Peyer’s patch, thus stimulating intestinal lymphatic transport and circumventing hepatic first-pass metabolism [82,86]. Furthermore, bile salts may act as permeation enhancers, which results in higher intestinal absorption and increased oral bioavailability of the encapsulated drug [82,86]. Additionally, skin permeability is also enhanced by bile salt-stabilized vesicles (“bilosomes”) due to their high flexibility and deformability, which enables them to penetrate as intact vesicles through the stratum corneum and provide efficient transdermal delivery [82,85,86].

Yang et al., developed hyaluronic acid (HA)-functionalized bilosomes (BLs) for targeted delivery of celastrol (**6**) to the inflamed arthritic joint via ligand-receptor interaction [87]. Hyaluronic acid (HA) is a major constituent of the extracellular matrix and the most abundant glycosaminoglycan in synovial fluid protecting articular cartilage. The regulatory functions of HA related to inflammation, cell migration, and angiogenesis are mediated by specific HA receptors, namely CD44 overexpressed on the surface of activated macrophages in the arthritic joints. HA@**6**-BLs showed good hemocompatibility with no cytolysis or coagulation after incubation with 2% erythrocyte suspension for 3 h [87]. Cellular uptake of bilosomes by RAW264.7 macrophages were superior to free **6** with higher uptake for the coated bilosomes due to CD44 receptor-mediated endocytosis, as proved by cellular uptake inhibition upon addition of monoclonal anti-CD44 antibody (HUTCH-1) [87]. Encapsulation in bilosomes provided in vitro sustained release and improved the pharmacokinetic profile of **6** in vivo in CFA mice (Table S5, Supplementary Materials) [87]. HA-coated bilosomes showed higher mean residence time in the blood stream compared with uncoated bilosomes due to reduced complement opsonization and MPS sequestration. Moreover, higher accumulation of **6** in the joint fluid of rats with adjuvant-induced arthritis was observed with HA@**6**-BLs compared with **6**-BLs and free form. The relative intra-articular bioavailability of HA-coated bilosomes was calculated as 800% compared with celastrol (**6**) solution while that of uncoated bilosomes was 480%, demonstrating the effective targetability of HA@**6**-BLs toward the inflamed joint [87]. The antiarthritic effect of the nanoformulations, evaluated in the CAIA mouse model, showed a superior therapeutic efficacy of the coated bilosomes in reducing joint swelling, pannus formation and serum levels of pro-inflammatory mediators, improving the arthritic score, which was attributed to the deformability of bilosomes and the specific affinity of HA@**6**-BLs to CD44 receptors overexpressed on inflamed tissues [87].

Ginsenoside CK (**11**) is a promising anti-arthritic agent for RA therapy due to its potent anti-inflammatory and immunomodulatory properties, but clinical translation has been hampered by its poor water solubility, low intestinal permeability, and P-gp efflux. Therefore, folic acid-targeted liposomes, presenting good stability in gastric and intestinal fluids and low cytotoxicity, have been developed for oral delivery of **11** (FA-LP-**11**) [88]. In vitro cellular uptake of FA-LP-**11** by LPS-activated macrophages was enhanced compared with LP-**11**, demonstrating that folate targeting was effective at increasing cell uptake via folate receptor (FR)-mediated endocytosis [88]. In rats with adjuvant-induced arthritis, FA-LP-**11** significantly inhibited the expression of pro-inflammatory cytokines, improved synovial hyperplasia, and reduced joint swelling, which improved arthritis scores [88].

#### 4.3.2. Niosomes

Niosomes are non-ionic surfactant-based vesicles, either unilamellar or multilamellar, structurally similar to liposomes but possessing higher physicochemical stability, improved shelf-life, and lower production cost [83]. Niosomes are formed by self-assembly of non-ionic surfactants and cholesterol in aqueous media. Formation of bilayer vesicles instead of micelles is dependent on the hydrophilic–lipophilic balance (HLB) of the surfactant, which is usually in the range 4–8, although cholesterol addition allows the use of surfactants with higher (more hydrophilic) HLB values [83,89]. Nonionic surfactants used in niosome preparation are mainly polyoxyethylene ethers (Brij^®^), sorbitan esters (Span^®^) and polyoxyethylene sorbitans (Tween^®^), which display a good safety profile. These surfactants may further act as solubilizers, emulsifiers, and permeation enhancers, as well as P-gp inhibitors, thus enhancing drug absorption and bioavailability [83,89].

Jamal et al. optimized niosomal gel formulations by experimental design for enhanced transdermal delivery of ursolic acid (**12**) in arthritis therapy [90]. Ex vivo permeation studies in rat skin revealed that the flux of niosomal formulation was enhanced compared with liposomal formulation of **12** used as control, which was attributed to eventual disruption of the densely packed lipids that fill extracellular spaces of the stratum corneum [90]. In mice with adjuvant-induced arthritis, the anti-arthritic efficacy of the formulations tested was in the order **12**-NF-gel > standard gel (positive control) > **12** oral suspension [90]. The poor solubility of **12** leading to poor absorption was responsible for the weak activity of the oral suspension. Furthermore, analgesic activity of **12**-NF-gel formulation was superior to that of the standard marketed gel of diclofenac/methyl salicylate (Omni gel) used as positive control due to enhanced skin penetration with gradual drug release over a longer period of time, without causing skin irritation [90]. Therefore, transdermal delivery of ursolic acid formulated as niosomal gel stands as a potential strategy for safe and efficient treatment of arthritis.

#### 4.3.3. Phytosomes

Phytosomes are lipid-based vesicles produced by complexation of bioactive phytochemicals with phospholipids (usually PC), often at 1:1 molar ratio, via hydrogen bonding between the active constituent and the lipid polar head group while the long fatty acyl chains wrap around the hydrophilic moieties of the complex, forming a lipophilic envelope [83,91]. The active constituent is thus an integral part of the phytosomal membrane (in marked contrast with liposomes, where it is distributed either in the membrane layers or in the aqueous compartment), which improves stability and bioavailability, allows for sustained release, and reduces drug leakage and toxicity. Phytosomes are characterized by a high bioactive phytochemical/lipid ratio when compared to liposomes and a high encapsulation efficiency since the drug is chemically conjugated with the phospholipid rather than physically entrapped [83,91].

Freag et al. developed self-assembled phospholipid-based phytosomal nanocarriers (**6**-PHY) to improve solubility and oral bioavailability of the anti-arthritic agent celastrol (**6**) [92]. The phytosomal formulation exhibited a sustained release profile in vitro compared with free **6** solution, which was attributed to the gradual dissociation of celastrol from the phospholipid complex and subsequent diffusion to the release medium (PBS pH 6.8) through the dialysis membrane. Pharmacokinetic studies in rabbits showed that **6**-PHY significantly enhanced oral absorption of **6** compared with free **6** solution, achieving a relative bioavailability of 410.7% [92].

Sharma et al. developed several vesicular formulations to enhance bioavailability and anti-inflammatory activity of boswellic acids (**2**, **3**, **8** and **9**) including phytosomes, liposomes, and niosomes, incorporated into 5% Carbopol 934 gel for topical application [93]. Phytosomes, prepared by complexation of boswellic acids with phosphatidylcholine (PC), were more effective than the other vesicular systems at reducing the inflammatory increase in paw volume in the carrageenan-induced rat paw oedema model, presumably due to enhanced absorption of the boswellic acids-PC complex through the skin [93]. The efficiency in oedema inhibition (%) at 5 h after carrageenan induction was in the order: phytosomes (88.89 ± 3.17%) > niosomes (79.63 ± 3.14%) > liposomes (77.78 ± 3.012%) > plain boswellic acid (68.52 ± 2.37%). The results highlighted the potential of phytosomal nanocarriers to improve oral bioavailability of hydrophobic triterpenoids, thus paving the way for their use in oral arthritis therapy [93].

Recently, Zhu et al. developed selenium-deposited celastrol (**6**) phytosomes (Se@**6**-PTs) to formulate two anti-inflammatory agents, celastrol and selenium, aiming to enhance the anti-arthritic effect of **6** via synergistic sensitization [94]. The formulation showed a sustained drug release both in acidic (HCl pH 1.2) and neutral (PBS pH 6.8) media compared with Se-free phytosomes (**6**-PTs). Both formulations exhibited faster drug release in acidic media due to dissociation of **6** from the phospholipid complex via proton exchange. However, Se@**6**-PTs showed slower drug release relative to **6**-PTs in acidic as well as in neutral media, suggesting that Se deposition/coating can reduce unwanted drug release in the gastrointestinal tract, avoiding intestinal P-gp efflux and improving oral bioavailability [94]. Se@**6**-PTs and **6**-PTs showed readily uptake by enterocytes (Caco-2 cells) due to high affinity of phospholipid to the biomembrane, which was only slightly masked by Se deposition. Internalization of the phytosomes occurred by the paracellular route, presumably due to the deformability and permeability of phytosomes prepared with sodium oleate and soy phosphatidylcholine [94]. Se@**6**-PTs showed enhanced anti-arthritic efficacy in rats with adjuvant -induced arthritis compared with **6**-PTs, attenuating inflammatory cell infiltration and pannus formation, reducing joint swelling and bone damage, and improving arthritic scores [94]. Moreover, Se@**6**-PTs were more effective at reducing serum levels of pro-inflammatory mediators (NO, TNF-α, and IL-6) than **6**-PTs, which was attributed to selenium’s sensitization to celastrol (**6**), which acts cooperatively to inhibit cytokine release [94].

**Table 4:** Vesicular drug delivery systems (VDDSs) employed to improve bioavailability and/or therapeutic effect of antiarthritic natural triterpenoids.

Triterpenoid/Drug Delivery System	Preparation Method	Characterization	Cell/Animal Model	Results	Ref.
Ginsenoside CK (**11**) Folate-targeted liposomes (FA-LP-**11**)	LP-**11** and FA-LP-**11** prepared by the ethanol injection method with lipid phase:water phase ratio 1:10 (*v*/*v*) using EPC/Chol/TPGS/**11** (32:16:8:7 mass ratio) or EPC/Chol/TPGS/DSPE-mPEG-FA/**11** (32:16:6.4:1.6:7:7 mass ratio), respectively, in ethanol	FA-LP-**11**: size 249.13 ± 1.40 nm, PDI 0.18 ± 0.03, ZP −4.60 ± 0.80 mV; EE 93.33 ± 0.05%. LP-**11**: size 221.10 ± 2.80 nm, PDI 0.14 ± 0.05, ZP −3.30 ± 0.27 mV; EE 94.46 ± 0.22%	LPS-activated macrophages (RAW264.7 cells). AA in male SD rats (n = 6)	Release studies using artificial gastric (pH 1.2) and intestinal (pH 6.8) juices showed a slow release of **11** from FA-LP-**11** in gastric juice (15% in 2 h vs. 30% for free **11**) and slowly in intestinal fluid (70% in 24 h vs. 96% for free **11**). FA-LP-**11** was shown to target activated macrophages in vitro and increase cell uptake via FR-mediated endocytosis compared to LP-**11**. In vivo, FA-LP-**11** reduced TNF-α, IL-1β, IL-2, and IL-6 in the serum of AA rats, improved synovial hyperplasia, inflammatory cell infiltration, and bone erosion.	[88]
Boswellic acids (mixture **2**, **3**, **8** and **9**) Liposomes	Lipid film hydration using soy PC and cholesterol at 7:3 molar ratio plus boswellic acids followed by incorporation into 5% Carbopol 934 gel.	Size 324.45 nm; EE 85 ± 4.09%	Carrageenan-induced hind paw oedema in Wistar rats (n = 6)	Liposomal formulation showed enhanced anti-inflammatory effect, inhibiting oedema by 77.78 ± 3.02% compared with 68.52 ± 2.37% for plain boswellic acids.	[93]
Celastrol (**6**) Selenium-deposited phytosomes (Se@**6**-PTs)	Melting-hydration followed by in situ reduction using soy PC (17 mg) and **6** (10 mg) at 1:1 stoichiometric ratio mixed with aqueous solution (10 mL) of sodium olate (25 mg), Na_2_SeO_3_ (10 mg) and excess of reduced GSH (which reduces Se^4+^ to Se that precipitates onto the surface of **6**-PTs)	Size 126 nm (106.9 nm for CEL-PTs), PDI 0.228, ZP −25 mV; EE 98.85%	Caco-2 cells. AA in male SD rats (n = 5)	In vitro release studies showed sustained release of **6** from Se@**6**-PTs both in acidic (HCl pH 1.2) and neutral (PBS pH 6.8) media compared with **6**-PTs. Phytosomes were shown to promote the transepithelial transport of **6** by the paracellular route in Caco-2 cells. Se@**6**-PTs and **6**-PTs were able to ameliorate joint inflammation, reduce joint swelling, decrease serum levels of NO, TNF-α and IL-6, and improve arthritic scores in AA rats. The in vivo anti-arthritic effect of Se@**6**-PTs was superior to **6**-PTs presumably due to Se enhancing the therapeutic efficacy of 6 synergistically.	[94]
Boswellic acids (mixture **2**, **3**, **8** and **9**) Phytosomes	Formation of complex between boswellic acids and soy PC at 1:1 molar ratio followed by phytosome formation by mixing boswellic acids-PC complex and cholesterol at 7:3 molar ratio. Phytosomes were then incorporated into 5% Carbopol 934 gel.	Size 508.32 nm.	Carrageenan-induced hind paw oedema in Wistar rats (n = 6)	Phytosomal formulation showed enhanced anti-inflammatory effect, inhibiting oedema by 88.89 ± 3.17% compared with 68.52 ± 2.37% for plain boswellic acids.	[93]
Ursolic acid (**12**) Niosome gel (UANF)	**12**-loaded niosomes prepared by film hydration using phospholipid (65 mg), cholesterol (12.3 mg), Span 60 (85 mg) and **12**; niosomal-loaded gel formulation obtained by adding Carbopol 934 (1% *w*/*w*), PEG-400 (15% *w*/*v*) and TEA (0.5% *w*/*v*).	Size 665.45 nm; EE 92.74%. Transflux 17.25 μg/cm^2^/h	AA in Albino Wistar rats (n = 6)	Transdermal niosomal gel of **12** using Carbopol as release controlling polymer showed enhanced skin permeability ex vivo compared with liposomal formulation and higher efficacy in paw oedema reduction in CFA mice than commercial standard gel (Omni gel 1%) used as positive control and **12** oral suspension. Gradual **12** release from the niosomal formulation provided additional analgesic activity for a prolonged period of time without skin irritation.	[90]
Boswellic acids (mixture **2**, **3**, **8** and **9**) Niosomes	Reverse evaporation method using Span 60 and cholesterol at 7:3 molar ratio plus boswellic acids followed by incorporation into 5% Carbopol 934 gel.	Size 246.12 nm; EE 89 ± 5.32%	Carrageenan-induced hind paw oedema in Wistar rats (n = 6)	Niosomal formulation showed enhanced anti-inflammatory effect, inhibiting oedema by 79.63 ± 3.14% compared with 68.52 ± 2.37% for plain boswellic acids.	[93]
Celastrol (**6**) Hyaluronic acid-functionalized bilosomes (HA@CEL-BLs)	Thin film hydration with drug/lipid ratio 1:10 using soy PC (80 mg), DOTAP (20 mg) and **6** (10 mg) hydrated with 10 mL of SDC solution (2 mg/mL), further coated with HA (10 mg) by electrostatic complexation with DOTAP.	Uncoated vesicles (**6**-BLs): size 95.3 nm, ZP 4.8 mV. Coated vesicles (HA@**6**-BLs): size 118.4 nm, ZP -34.2 mV. DL 8.15% EE 99.56%	Macrophages (RAW264.7 cells). AA in SD rats (n = 6). CAIA in DBA/1 mice (n = 6)	The nanoformulation provided sustained release of **6** in vitro. The HA@**6**-BLs displayed high cellular uptake by macrophages (RAW264.7 cells) and targeted delivery efficiency for **6**, enhancing both systemic and intra-articular bioavailability of the triterpenoid in AA rats compared with non-targeted bilosomes and celastrol solution. Bilosomal formulations reduced joint swelling, inflammatory cell infiltration and serum levels of NO, TNF-α and IL-6 in the CAIA mouse model, improving arthritic scores, with HA@**6**-BLs showing enhanced therapeutic effect due to specific affinity toward CD44 receptors overexpressed on activated macrophages.	[87]

### 4.4. Self-Emulsifying Drug Delivery Systems

Self-emulsifying drug delivery systems (SEDDS) are isotropic mixtures of lipid (oil), surfactant and co-surfactant which spontaneously emulsify to produce fine oil-in-water (o/w) emulsions upon contact with aqueous media (e.g., gastrointestinal fluids) under mild agitation (e.g., gastrointestinal motility). SEDDS encompass both self-microemulsifying drug delivery system (SMEDDS) and self-nanoemulsifying drug delivery systems (SNEDDS), which produce microemulsions and nanoemulsions, respectively [95,96]. These lipid-based formulations can incorporate hydrophobic drugs, including bioactive triterpenoids, through solubilization in the lipid phase, and the drug-containing oily droplets formed upon in situ self-emulsification possess small size (usually below 100 nm) and high surface area, which promotes absorption by the intestinal epithelia [95,96]. Lipid digestion aided by bile secretion in the duodenum forming bile salt mixed micelles that incorporate the hydrophobic drug further contribute to drug solubilization and absorption [95,96]. Moreover, inclusion of long-chain and unsaturated lipids in SEDDS composition stimulates the formation of lipoproteins and chylomicrons, which promotes intestinal lymphatic transport, thus circumventing hepatic first-pass metabolism and increasing drug bioavailability following oral administration [95,96]. Surfactants present in SEDDS also contribute to the improvement in drug absorption by enhancing intestinal drug permeability, inhibiting P-gp-mediated efflux, and hindering the activity of intestinal CYP3A4 enzyme involved in phase I metabolism [95,96].

An adequate selection of lipid, surfactant, co-surfactant, and their ratios is crucial for the development of efficient SEDDS formulations, influencing drug loading and encapsulation efficiency as well as the type of emulsion, droplet size, and stability to environmental stresses (e.g., pH, temperature, and ionic strength) after dispersion. Regarding the lipid phase, (biocompatible and biodegradable) medium- and long-chain triglycerides from natural edible oils or their semisynthetic derivatives, with varying saturation degrees, are usually employed [95,96]. Surfactants, which adsorb at the oil-water interface and thus lowering interfacial tension, avoid coalescence of the oil droplets and are essential for SEDDS stabilization after dispersion [95,96]. Higher emulsification (leading to o/w droplets of smaller (nano) size) is achieved with surfactants possessing HLB > 12, such as hydrophilic non-ionic surfactants (e.g., Gelucire^®^, Cremophor^®^ and Labrasol^®^), at the concentration range 30–60% (*w*/*w*) [95,96]. Despite the good safety profile of non-ionic surfactants, large surfactant concentrations in SEDDS formulations, often necessary for efficient emulsification and drug solubilization, can irritate the wall of the gastrointestinal tract.

The addition of a co-surfactant/co-solvent enhances drug solubilization and dispersibility of the hydrophilic surfactant in the lipid phase, which allows the reduction of surfactant concentration in the formulation to prevent gastric irritation [95,96]. The co-surfactant further reduces interfacial tension, promotes smaller droplet size, and enhances fluidity of the interfacial film, thus avoiding the formation of liquid crystals [95,96]. Hydrophilic solvents suitable for oral administration, such as ethanol, propylene glycol, and PEG can be used as co-solvents [95,96], but their fast release from SEDDS upon dispersion decreases drug solubility within the oily droplets and can lead to drug precipitation.

SEDDS are liquid lipid formulations and require encapsulation in hard or soft gelatine capsules to improve storage stability and avoid irreversible precipitation of drug or excipients, yet interaction between the filling and the capsule shell has been noted. However, liquid SEDDS can be converted into solid dosage forms, for example, by adsorption onto solid carriers, freeze-drying, spray-drying, or hot melt extrusion. Solid SEDDS combine the enhanced drug solubility and bioavailability of liquid SEDDS with improved stability, controlled drug release, low production cost, and better patient compliance [95,96].

Xi et al. developed SNEDDS formulations to improve oral delivery and bioavailability of oleanolic acid (**13**) [97], composed of Sefsol^®^ 218 as lipid phase, Cremophor^®^ EL and Labrasol^®^ as surfactant mixture and Transcutol^®^P as cosurfactant at weight ratios of 50:25:25:0, 50:22.5:22.5:5, 50:20:20:10 and 50:17.5:17.5:15 loaded with oleanolic acid (20 mg/g). The formulation design and optimization were based on solubility studies, screening of excipients and construction of (pseudo)ternary phase diagrams that allowed the identification of self-emulsification regions [97]. The surfactant/cosurfactant ratio was determinant for droplet size and emulsification rate. The droplet size of the nanoemulsion was found to decrease with the increase of surfactant/cosurfactant weight ratio, which was attributed to the increased stability and condensation of the interfacial film provided by the surfactant [97]. The formulation without cosurfactant showed the smallest droplet size and polydispersity according to the transmission electron microscopy data, which was selected for the in vivo bioavailability studies. When compared with the commercial **13** tablet, this optimized SNEDDS formulation showed a higher dissolution rate in simulated gastric fluid, a 2.4-fold increase in relative bioavailability after oral administration to rats, and an increased mean retention time of oleanolic acid in the rat plasma (Table S5, Supplementary Materials) [97].

Yang et al. developed a SMEDDS formulation to improve solubility and oral bioavailability of oleanolic acid (**13**) [98]. Among the different oils, surfactants, and cosurfactants tested, solubilization of **13** was efficiently achieved in ethyl oleate, oleic acid, Labrasol^®^, Cremophor^®^EL, and ethanol. The high solubilization capacity of the vehicles is necessary for complete drug dissolution in order to avoid the precipitation of the drug upon dilution in the gastrointestinal tract. However, oils and surfactants with good solubility do not necessarily possess a good emulsifying capacity, and compatibility tests were performed by mixing different surfactants and oils at different surfactant/oil ratios (90:10, 80:20 and 70:30). Based on the results of the compatibility tests, ethyl oleate and Cremophor^®^ EL, which showed the best emulsifying capability, were chosen as the optimum oil and surfactant, respectively [98]. Selection of ethanol as the optimal cosurfactant was based on pseudoternary phase diagrams of surfactant/cosurfactant, oil, and water constructed using the titration method. The mixed surfactant was prepared at a fixed surfactant/cosurfactant weight ratio (2:1) while the oil/mixed surfactant ratio varied from 9:1 to 1:9. Each mixture was titrated with water and the concentration of water at which turbidity-to-transparency and transparency-to-turbidity transitions occurred was derived from the weight measurements and the values used to determine the boundaries of the microemulsion domain [98].

A ternary phase diagram of surfactant, cosurfactant, and oil for formulation optimization was constructed from mixed surfactants prepared at different surfactant/cosurfactant ratios which were further mixed with oil at weight ratios of oil/mixed surfactant of 9:1, 8:2, 7:3, 6:4, 5:5, 4:6, 3:7, 2:8, and 1:9 [98]. Each mixture was 100-fold diluted with purified water at 37 °C under gentle stirring and the in vitro performance of the formulations was visually evaluated according to the grading system. The optimized formulation, composed of Cremophor^®^EL, ethanol, and ethyl oleate at a weight ratio of 50:35:15 allowed for sustained release of **13** in vitro as determined using a dialysis method [98]. **13**-loaded SMEDDS exhibited an improved pharmacokinetic profile in rats when compared with the commercial tablet, achieving a 5.07-fold increase in oral bioavailability (Table S5, Supplementary Materials) [98]. Several factors may contribute to the improved bioavailability of the SMEDDS formulation: (i) in the gastrointestinal tract, SMEDDS forms a fine o/w microemulsion with a droplet size below 100 nm and large interfacial area, thus promoting drug absorption; (ii) the high surfactant concentration in SMEDDS may enhance the permeability of the intestinal epithelium; (iii) the lipid in the SMEDDS stimulates the formation of lipoproteins and chylomicrons, thus promoting absorption through the lymphatic pathway and circumventing first-pass metabolism [98]. 

Qi et al. prepared solid self-microemulsifying dispersible tablets of celastrol (**6**) by wet granulation compression technique using microcrystalline cellulose KG 802 as the solid adsorbent [99]. The solid carrier was added to an optimized liquid **6**-SMEDDS formulation composed of 25% ethyl oleate (lipid), 60% octyl polyethylene glycol phenyl ether, OP-10 (surfactant), and 15% Transcutol^®^P (co-surfactant) with 10% (*w*/*w*) **6** content. Transmission electron microscopy (TEM) images showed that the droplet size of the reconstituted microemulsion (23.17 ± 0.86 nm) was not significantly different from that of liquid **6**-SMEDDS (22.05 ± 1.56 nm), indicating that the solidification process did not affect the microemulsion droplet size [99]. In vivo pharmacokinetic studies showed that the relative bioavailability of **6**-SMEDDS and SMEDDS dispersible tablets was 569 ± 7.07% and 558 ± 6.77%, respectively, compared with **6** suspension in 0.4% sodium CMC, after oral administration to rats at equivalent **6** dose (4 mg/kg) [99]. There was no significant difference between the liquid and solid SMEDDS formulations (Table S5. Supplementary Materials), indicating that the self-microemulsifying ability was well maintained in the **6**-SMEDDS dispersible tablets [99].

### 4.5. Solid Lipid Nanoparticles and Nanostructured Lipid Carriers

Solid lipid nanoparticles (SLNs) are spheroidal nanostructures comprising a solid lipid core stabilized by an outer surfactant layer. The lipid matrix is composed of solid lipid or mixture of solid lipids (0.1–30%) that remain solid both at room and body temperature, forming an almost perfect crystalline structure. Thus, lipids with high melting points with well-ordered crystalline structure and stable polymorphic form are preferred to ensure that the NPs remain solid at physiological temperature and avoid polymorphic transitions due to temperature changes. The selected lipids should be biocompatible, resist oxidation and degradation, and show compatibility with the encapsulated drug. Physiological lipids or lipids used as common pharmaceutical ingredients, such as fatty acids, (tri)glycerides, fatty alcohols, and waxes, are commonly employed in the fabrication of SLNs. Stabilizing agents (0.5–5% *w*/*w*) include non-ionic surfactants (polysorbates and poloxamers), lecithin and bile salts, usually regarded as safe (GRAS), which act as emulsifiers to reduce the interfacial tension between the lipid and aqueous phases during the manufacturing process. Surfactant selection is mainly based on emulsifying ability, biocompatibility, and compatibility with the lipid matrix (to prevent phase separation) according to the surfactant HLB value. SLNs can be produced by several methods that do not require the use of organic solvents, such as high-pressure homogenization, ultrasonication, supercritical fluid technology, double emulsion, or phase inversion temperature methods [100]. The lipid/surfactant ratio influences the size and stability of the NPs, drug loading and controlled release. Higher lipid content can enhance drug encapsulation but can also result in slower drug release rates. Formulation optimization requires experiments with different lipid/surfactant ratios in order to obtain stable NPs with the desired particle size and control of drug release kinetics. Thus, physicochemical properties of SLNs, such as size, polydispersity, surface charge, stability, drug loading and release profile can be modulated by appropriate selection of lipid, surfactant, and SLN composition [100].

SLNs can incorporate both hydrophobic and hydrophilic drugs in the lipid matrix, however, efficient drug delivery is hampered by limited drug loading due to lipid crystalline nature, gelation tendency, and drug expulsion during storage caused by lipid polymorphism. This can be avoided by reducing the degree of crystallinity of the solid lipid core through blending with low amounts of liquid lipid (oil), leading to a second generation of lipid NPs called nanostructured lipid carriers (NLCs), with improved stability and drug loading capacity due to a less structured (more flexible) lipid matrix that provides more space for drug dissolution and payload [101]. Furthermore, the solid/liquid lipids ratio in NLCs can be adjusted to modulate the structure and crystallinity of the lipid matrix as well as for optimization of drug loading and release profile. Drug immobilization in the solid lipid matrix of SLNs and NLCs protects labile drugs from chemical and enzymatic degradation, avoids drug leakage, and contributes to sustained drug release, enabling administration by parenteral and non-parenteral routes [102].

Lipid NPs, being composed of lipids structurally similar to those found in rich-fat food, are prone to digestion by lipases and co-lipases after oral administration, similarly to ingested dietary lipids [103]. The resulting lipolytic products, mainly fatty acids and monoglycerides, are subsequently converted into mixed micelles for absorption by enterocytes upon interaction with bile salts in the small intestine, which can prevent precipitation of the encapsulated lipophilic cargos [103]. The enhanced oral absorption of encapsulated drugs in lipid NPs has been attributed to the lipolysates that contribute to the prolongation of residence time in the gastrointestinal tract, which promotes bile secretion and stimulates intestinal lymphatic transport [103], thus improving the bioavailability of hydrophobic drugs, including the antiarthritic triterpenes. Lipid structure and drug lipophilicity influence the absorption process: long-chain triglyceride lipids and highly lipophilic encapsulated drugs preferentially bind with intestinal proteins and transport through lymphatic system while short- or medium-chain lipids and amphiphilic encapsulated drugs reach the systemic circulation through the portal vein [103]. Moreover, the surfactants employed in these formulations may act as permeation enhancers, promoting absorption in the intestinal epithelium and across the skin, depending on the administration route. NLCs are particularly suited for transdermal delivery due to their small size and good biocompatibility, forming a monolayer on skin upon topical application that prevents transepidermal water loss. Skin hydration may open intergaps between corneocytes that facilitate drug penetration into the skin.

Lingling et al. developed SLNs using glycerin monostearate (GMS) as lipid and poloxamer 188 as surfactant in order to improve oral bioavailability of asiatic acid tromethamine salt (**14**) [104]. DSC and X-ray analyses of the formulation showed that **14** was in amorphous state while the crystal degree of GMS significantly decreased. Moreover, in vivo pharmacokinetic studies in rats showed that the bioavailability of the asiatic acid salt (**14**) when encapsulated in SLNs was 2.5-fold higher compared to free asiatic acid salt (Table S5, Supplementary Materials) after a single oral dose in rats [104].

Zhou et al. developed an optimized NLC formulation for oral delivery of celastrol (**6**) using stearic acid and isopropyl myristate as the solid and liquid lipid matrices, respectively, mixed at 3:1 weight ratio. The lipid NPs were prepared by the solvent evaporation method using 10% soybean lecithin and 10% TPGS as emulsifiers and loaded with 4% *w*/*w* of **6** [105]. The average particle size, zeta potential, drug loading and entrapment efficiency of the optimized NLCs were 109.6 ± 5.8 nm, −29.8 ± 1.3 mV, 3.93 ± 0.02%, and 78.64 ± 0.37%, respectively [105]. **6**-NLCs exhibited a delayed release profile in vitro due to the mixing of drug diffusion and lipid matrix erosion. Differential scanning calorimetry (DSC) showed that **6** was not in a crystalline state but in an amorphous state in the NLC matrix [105]. 

Absorption studies using the rat intestinal perfusion model showed that the effective permeability (*P*_eff_*) of **6** solution was higher in the duodenum and jejunum than in the ileum and colon, and the very low values (*P*_eff_* < 1) obtained suggest poor intestinal absorption of **6** [105]. The effective permeability was significantly enhanced by NLCs, especially in the jejunum and duodenum, with *P*_eff_* values for **6**-NLCs being 2.7 and 2.1-fold higher, respectively, than those of **6** solution. The efficacy of NLCs in enhancing intestinal absorption of **6** was attributed to improved drug solubility while the small particle size (109.6 ± 5.8 nm) combined with the large surface area of the NLCs increased the contact area of the NPs with the rat intestinal epithelium, promoting **6** uptake by transcellular and/or paracellular pathways [105]. Furthermore, the lipid components of the NLCs, being structurally similar to dietary lipids, may induce bile secretion in the small intestine and associate with bile salt to form mixed micelles that contribute to the enhanced absorption of **6**-NLCs in the rat intestine. The use of the TPGS surfactant in the NLC formulation also contributed to the improved absorption since TPGS can act as a permeation enhancer and increase the intestinal epithelial permeability by disturbing the cell membrane and reversibly opening the tight junction of intestinal epithelial cells [105].

According to in vitro cell viability studies (MTT assay) using human Caco-2 cells as a model of the intestinal epithelial cell barrier, decreased cytotoxicity was observed for celastrol loaded-NLCs (IC_50_ 2.11 μg/mL) when compared with celastrol solution (IC_50_ 0.34 μg/mL) [105]. Cell viability rates in the presence of blank NLCs were higher than 90%, showing that the NPs had no significant in vitro cytotoxicity due to the good biocompatibility of the NLC lipid and surfactant components, which are FDA approved pharmaceutical excipients. In the NLC manufacturing process, the use of the TPGS surfactant contributed to high entrapment efficiency, being able to reduce drug leakage from the oily droplets [105]. The long hydrophilic PEG chain and the bulky lipophilic tocopherol succinate contribute to the large surface area of TPGS responsible for improved drug encapsulation efficiency and efficient protection of encapsulated hydrophobic celastrol from diffusion or partition to external phase, thus reducing drug cytotoxicity.

Zhang et al. prepared **6**-loaded lipid nanospheres (**6**-LNs) with a mean size of 150 nm composed of soybean lecithin, soybean oil, and sodium oleate [106]. **6**-LNs were stable in fasted-state simulated intestinal fluid (pH 6.5) containing lipase due to their tightly assembled structure resulting from the fine miscibility of **6** with the lipid materials, as seen in FTIR spectra, and the coating of hydrophilic sodium oleate isolating the lipid matrix from the active site of the enzyme [106]. The pharmacokinetic study in rats showed that absolute bioavailability of **6** suspension and **6**-LNs were 13.35% and 30.01%, respectively, while the relative bioavailability of **6**-LNs compared with **6** suspension achieved 224.88%, demonstrating the capacity of LNs to significantly enhance celastrol oral bioavailability [106]. Further mechanistic studies employing in situ single-pass rat intestinal perfusion assay revealed that simivastasin, a nonspecific caveolin-mediated transport inhibitor, and cycloheximide, a chylomicron formation inhibitor that blocks lymphatic transport, significantly reduced **6**-LNs absorption [106]. Therefore, the increase in oral bioavailability of celastrol upon encapsulation in LNs was attributed to enhanced intestinal permeability via nonspecific caveolae-dependent endocytosis (a pathway involved in the absorption of dietary fatty acids like sodium oleate present in the nanocarrier composition) and promotion of lymphatic transport, circumventing the first-pass effect both in gut and liver [106]. 

Chen et al. developed cell-penetrating peptide (CPP)-coated NLCs to improve oral absorption of **6** [107]. Cell-penetrating peptides (CPPs) are short cationic amphiphilic peptides rich in basic amino acids (e.g., arginine and lysine), which are mainly absorbed via translocation and endocytosis (clathrin-mediated, caveolae-mediated, and micropinocytosis) pathways, being able to transport bioactive small molecules as well as macromolecules across cell membranes. The CPP-**6**-NLCs exhibited a controlled release profile under in vitro under physiological conditions (PBS pH 7.4, 37 °C) and reduced celastrol toxicity against intestinal Caco-2 cells in the MTT assay [107]. Using an in-situ rat intestinal perfusion model, the authors showed that the absorption of **6** from CPP-**6**-NLCs in the rat duodenum and jejunum was significantly increased compared with celastrol solution and also uncoated nanoparticles (**6**-NLCs) [107]. CPP-**6**-NLCs showed an improved pharmacokinetic profile in beagle dogs compared with **6**-NLCs and **6** solution, achieving relative oral bioavailability of 149.91% and 484.75%, respectively (Table S5, Supplementary Materials) [107]. The high positive charge density of the CPP used (Ste-R_6_L_2_), mainly composed of arginine sequences, promotes electrostatic interaction with negatively charged cell membrane which might facilitate endocytosis and direct penetration of CPP-coated NPs across the intestinal epithelium upon oral administration [107].

Kang et al. developed a transdermal delivery system of NLCs, loaded with both celastrol (**6**) and indomethacin (**6**-Indo-NLCs) and incorporated into Carbopol gel, to combine the anti-arthritic activity of celastrol with the anti-inflammatory and analgesic effects of indomethacin [108]. In vitro skin permeation studies showed sustained transdermal release from **6**-Indo-NLCs gel across excised rat skin [108]. Visualization of transdermal translocation of **6**-Indo-NLCs in vivo was achieved by confocal laser scanning microscopy, confirming their presence on the surface of viable epidermis and dermis at 1 h, which was attributed to the small particle size (~27 nm) and sustained release from the gel nanoformulation [108]. The results also revealed that NLC were able to penetrate across the skin as intact vesicles while some entered the skin through the hair follicle [108]. Transdermal delivery of **6**-Indo-NLCs gel was very effective at decreasing serum levels of IL-1β, TNF-α, β-endorphin and substance P, reducing paw oedema, synovial inflammation and pain in arthritic rats without causing skin irritation after long-term administration [108]. Combination therapy was more efficient than the formulation of either drug alone, and anti-arthritic activity increased in the order Indo-NLCs gel < **6**-NLCs gel < **6**-Indo-NLCs gel [108]. Toxicological studies showed no gastrointestinal, kidney, or reproductive toxicity after topical administration of **6**-Indo-NLCs gel, presumably due to the biocompatible nature of the nanocarrier [108].

**Table 5:** Self-emulsifying drug delivery systems (SEDDSs), lipid nanoparticles (SLNs + NLCs), and inorganic silica NPs employed to improve bioavailability and/or therapeutic effect of antiarthritic natural triterpenoids.

Triterpenoid/Drug Delivery System	Preparation Method	Characterization	Cell/Animal Model	Results	Ref.
*Self-emulsifying drug delivery systems (SEDDSs)*					
Oleanolic acid (**13**) Self-nanoemulsifyed drug delivery system (SNEDDS)	Prepared by mixing Sefsol^®^218 (lipid) with Cremophor^®^EL/Labrasol^®^ (surfactant mixture) at 50:25:25 *w*/*w*, then adding **13** (20 mg/g)	Size 38.4 ± 0.2, PDI 0.055	Male SD rats (n = 5)	Higher dissolution rate in simulated gastric fluid and 2.4-fold higher bioavailability after oral administration to rats compared with suspension of commercial **13** tablets. Improved PK profile compared with commercial **13** tablets.	[97]
Oleanolic acid (**13**) Self-microemulsifying drug delivery system (SMEDDS)	Prepared by mixing Cremophor^®^EL (surfactant), ethanol (co-surfactant) and ethyl oleate (lipid) at 50:35:15 *w*/*w* with gentle stirring at 40 °C, then adding **13** (1% *w*/*w*)	Droplet size 49.7 nm DL 13.196 ± 0.328 mg/g	Male SD rats (n = 5)	SMEDDS allowed for sustained release of **13** at physiological temperature (37 °C) in vitro. **13**-SMEDDS showed improved PK profile and 5.07-fold increase in oral bioavailability.	[98]
Celastrol (**6**) Solid and liquid SMEDDS	Liquid SMEDDS prepared by mixing ethyl oleate (lipid), OP-10 (surfactant) and Transcutol^®^P (co-surfactant) at 25:60:15 *w*/*w*, then adding **6** (10% *w*/*w*); Solid SMEDDS dispersible tablets prepared by wet granulation compression method using MCC KG 802 as solid adsorbent	Liquid SMEDDS: droplet size 23.17 ± 0.86, PDI 0.104 ± 0.009 Solid SMEDDS: droplet size 22.05 ± 0.86 1.56, PDI 0.113 ± 0.011	Male SD rats (n = 6)	TEM images showed that the solidification process did not affect morphology and droplet size of the self-microemulsifying system. SMEDDS dispersible tablets and liquid SMEDDS significantly increased solubility and bioavailability of **6** after oral administration to rats (4 mg/kg) when compared with **6** suspension at the same dosage.	[99]
*Lipid nanoparticles (SLNs + NLCs)*					
Asiatic acid tromethamine salt (**14**) SLNs	Modified solvent injection method using 10 mg GMS (solid lipid) and **14** in 1.07 mL ethanol (oil phase) and 0.1 g poloxamer P188 (surfactant) in 20 mL water (aqueous phase); freeze-dried powder obtained by lyophilization using 10% lactose as cryoprotectant.	Size 237.6 ± 3.4 nm, ZP −35.9 ± 0.14 mV, DL 31.9 ± 2.9%, EE 64.4 ± 5.9%	Male SD rats (n = 6)	Bioavailability 2.5-fold higher compared with free **14**; good storage stability with no coacervation at pH 1.2. Improved PK profile compared with the free salt.	[104]
Celastrol (**6**) + Indomethacin NLCs	Emulsification evaporation-solidification method using **6** (10 mg), Indomethacin (10 mg), Precirol^®^ATO-5 (solid lipid), Labrasol^®^ALF (liquid lipid) at 70:30, Cremophor^®^RH40 (surfactant), then adding 0.5% *w*/*v* Carbopol 940 to produce hydrogel	Size 26.92 ± 0.62 nm, DL 3.65 ± 0.05%, EE 96.56 ± 1.36%	AA in male SD rats (n = 6)	Ex vivo permeation studies showed sustained transdermal drug release from 6-Indo-NLCs gel across excised rat skin. The nanocarriers were able to translocate across intact rat skin in vivo reaching viable epidermis and dermis as intact vesicles due to their small size and also hair follicle pathway. 6-Indo-NLCs gel was safe and caused no skin irritation. Arthritic rats treated with 6-Indo-NLCs gel showed decreased levels of IL-1β, TNF-α, β-endorphin and substance P, reduced paw oedema, synovial inflammation, and pain. Combination therapy was more effective than either drug alone, and anti-arthritic effect decreased in the order Indo-NLCs gel < 6-NLCs gel < 6-Indo-NLCs gel.	[108]
Celastrol (**6**) CPP-coated NLCs (CPP-Cel-NLCs)	Solvent evaporation method using Precirol^®^ATO-5 (360 mg) as solid lipid and Labrafil^®^M 1944CS (120 mg) as liquid lipid at 3:1 *w*/*w*, SLT (60 mg) and TPGS (60 mg) as emulsifiers, **6** (30 mg) and 0.5% *w*/*w* F68 as surfactant. **6**-NLCs coated with CPP (30 mg) at drug/CPP ratio 1:1 by electrostatic complexation	Uncoated (**6**-NLCs): size 102.4 ± 12.8 nm, PDI 0.129 ± 0.028, ZP −26.2 ± 2.71 mV, EE 74.04 ± 0.87%. Coated (CPP-**6**-NLCs): size 126.7 ± 9.2 nm, PDI 0.142 ± 0.03, ZP 28.7 ± 3.4 mV, EE 72.64 ± 1.37%.	Caco-2 cells. Male SD rats (n = 4). Beagle dogs (n = 6).	**6** toxicity in Caco-2 cells (IC50 0.316 ± 0.037 μg/mL) was reduced by encapsulation in uncoated **6** -NLCs (IC50 1.171 ± 0.098 μg/mL) and coated CPP-**6** -NLCs (IC50 0.885 ± 0.094 μg/mL). Cellular uptake was enhanced for coated compared with uncoated NLCs. CPP-**6** -NLCs and **6**-NLCs exhibited a controlled release profile in vitro under physiological conditions (PBS pH 7.4, 37 °C) with 40.41% and 44.84% celastrol release after 24 h, respectively. In the in situ rat intestinal perfusion assay, absorption of celastrol from CPP-**6**-NLCs in rat duodenum and jejunum was increased when compared with celastrol solution and uncoated nanocarriers (**6**-NLCs). CPP-**6**-NLCs showed improved pharmacokinetic profile in beagle dogs compared with uncoated **6**-NLCs and NLC suspension, achieving relative oral bioavailability of 149.91% and 484.75%, respectively.	[107]
*Inorganic silica NPs*					
Celastrol (**6**) pH-responsive hollow MSNs (CEL@HMSNs-Cs)	Solid silica NPs (500 mg) (core) coated with mesoporous silica (shell) using TEOS (1.5 mL) and CTAB (0.75 mg) as stabilizer. Selective etching of the core by aqueous Na_2_CO_3_ (6 mol/L, 50 mL) at 80 °C produced hollow MSNs (100 mg), loaded with **6** (15 mg/mL, 10 mL) by passive diffusion and coated with chitosan (10% *v*/*v*) using GPTMS (100 mg) as cross-linker.	Coated (**6** @HMSNs-Cs): mean size 290.17 nm, ZP 19.9 ± 0.7 mV, DL 24.3%; uncoated (**6** @HMSNs): mean size 260.76 nm, ZP −9.5 ± 0.7 mV, DL 28.2%. BET pore diameter, pore volume (*V*_pore_) and specific surface area (*S*_BET_) of empty MSNs (HMSNs) were 2.4 nm, 0.7668 cm^3^/g and 1006.8 m^2^/g, respectively, decreasing after drug loading (*V*_pore_ 0.2476 cm^3^/g and *S*_BET_ 235.13 m^2^/g) and upon coating (*V*_pore_ 0.1205 cm^3^/g and *S*_BET_ 52.816 m^2^/g)	IL-1β stimulated rat chondrocytes; MIA-induced knee OA in male SD rats (n = 6)	The pH-responsive release profile of the nanoformulation, conducted in different buffers simulating physiological (pH 7.7) and osteoarthritic (pH 6.0) conditions, showed cumulative **6** release of 21.7% and 68.9%, respectively, after 150 min. **6**@HMSNs-Cs decreased the expression levels of IL-1β, TNF-α, IL-6, MMP-3 and MMP-13 up-regulated in IL-1β-stimulated rat chondrocytes by inhibiting the NF-κB pathway. In rats with MIA-induced knee OA, intra-articular injection of **6**@HMSNs-Cs increased paw withdrawal threshold and ameliorated articular surface erosion and joint effusion.	[109]

### 4.6. Inorganic Silica Nanoparticles

Mesoporous silica nanoparticles (MSNPs), with pore sizes in the range 2–50 nm, are versatile inorganic nanocarriers due to high specific surface area, large pore volume, tunable pore aperture, good biocompatibility, tunable biodegradability, and easy surface chemistry modification [110]. Preferential drug adsorption to the MSNPs results from electrostatic, hydrogen-bonding, and van der Waals interactions between the drug and the MSNP internal surface, contributing to the high drug loading capacity of these nanocarriers [110]. In hollow MSNPs, with tunable hollow cores and mesoporous shells, the drug can be efficiently accommodated both in the mesoporous channels and in the interstitial hollow cavities, thus enhancing drug loading capacity [111]. Tunable pore aperture can be achieved by surface functionalization with stimuli-responsive gatekeepers for controlled drug release [111]. 

Jin et al. developed pH-responsive hollow mesoporous silica nanoparticles (HMSNs) capped with chitosan (Cs) for intra-articular delivery of celastrol (**6**) in knee osteoarthritis [109], taking advantage of the acidic milieu of the synovial fluid in (osteo)arthritic joints. The chitosan coating exhibits an insoluble gel-like structure in alkaline media, providing a protection layer that reduces drug leakage. In an acidic environment, protonation improves water solubility, and the chitosan layer swells, exposing pore entrances and triggering drug release. Moreover, biodegradation of chitosan yields chondroprotective glucosamine, turning chitosan into an ideal gatekeeper for intra-articular administration in (osteo)arthritis therapy. The pH-responsive release profile in buffers of different pH, simulating physiological (pH 7.7) and osteoarthritic (pH 6.0) conditions, revealed that the nanoformulation was stable in alkaline media while in acidic media 68.9% of **6** was released in 150 min. In vitro studies showed that **6**@HMSNs-Cs decreased the up-regulated expression levels of IL-1β, TNF-α, IL-6, MMP-3, and MMP-13 in IL-1β-stimulated rat chondrocytes by inhibiting the NF-κB pathway [109]. In the MIA induced knee osteoarthritis rat model, intra-articular injection of **6**@HMSNs-Cs increased paw withdrawal threshold and ameliorated articular surface erosion and joint effusion [109].

## 5. Conclusions

The anti-inflammatory and anti-arthritic effects of many triterpenes, and their potential use as pharmacological leads and/or drug candidates for arthritis treatment require an appropriate pharmacokinetic profile and good bioavailability. Many triterpenes have poor water solubility with weak absorption after oral administration, are metabolised by liver enzymes such as cytochrome P450, and are mostly involved in phase I oxidation reactions. Pharmacokinetic and metabolism studies are critical for clinical drug development concerning the efficacy and safety of the drug candidate.

FDA-approved nanotechnology-driven products have increased over the past 30 years. These products include polymeric nanoparticles, micelle nanoparticles, liposome formulations, and nanocrystals, among others, that are used in conjunction with biologics or drugs. In the last decade, several nanocarriers, such as polymeric nanoparticles and polymeric micelles, have been developed to improve solubility, oral bioavailability, and the pharmacokinetic profile of bioactive triterpenoids, aiming to achieve a sustained release profile and to enhance the targeted delivery of anti-inflammatory triterpenoids for arthritis therapy.

Most studies of nanoformulations targeting arthritis have focused on rheumatoid arthritis, highlighting celastrol (**6**) as one of the triterpenoid compounds with promising potential to be a drug candidate. The various DDS comprising celastrol have been developed to reduce organ toxicity and promote selective drug delivery. Nanocarriers such as enzyme-responsive nanoparticles (PRNPs) made up of RGD-modified PLGA nanoparticles (RNPs) covered with MMP-9-cleavable PEG chains enable celastrol to decrease the number of osteoclasts and inflammatory macrophages in rat models with RA, as well as promoting high cellular uptake in RA human synovial macrophages and osteoclasts resulted from patients undergoing joint replacement surgery. Another interesting polymeric nanocarrier for celastrol is the human serum albumin (HSA)-Kolliphor^®^ HS15, which allows the in situ release of the compound to inhibit pro-inflammatory cytokine secretion, improving the therapeutic efficacy and safety profile. Also noteworthy are the polymeric micelles loaded with celastrol, such as PEGylated bilirubin self-assembled nanoparticles (BRNPs) and amphiphilic block copolymers based on polypropylene sulfide and poly(ethylene glycol) (PEPS), both of which reduce the levels of pro-inflammatory cytokines in macrophages and bone erosion. Moreover, very promising results concerning the arthritic effect and the arthritic score in the CAIA mouse model were found with hyaluronic acid-functionalized bilosomes loaded with celastrol. The marked flexibility and deformability of these vesicles to targeted delivery of celastrol also improved the pharmacokinetic profile of the compound in vivo in CFA-induced arthritis in mice.

Studies involving the encapsulation of other anti-arthritic triterpenoids (considering RA, OA, and gout) such as glycyrrhizin, ginsenosides, or boswellic acids, using diverse nanosized carriers have pointed to effective inhibition of transcriptional factors NF-κB, NLRP3 inflammasome, pro-inflammatory cytokines expression and biological markers for inflammatory arthritis, thus improving the pharmacological action and bioavailability, as well as a better-controlled release of the studied triterpenes.

Despite the promising therapeutic potential of the nanoDDSs, the toxicological issues of nanoparticles are still on the agenda of the in vivo biological data. In fact, the clinical translation requires further studies regarding NPs’ toxicity before implementing anti-arthritic triterpenoid nanoformulations. A critical concern for clinical translation of nanoformulations is the eventual toxicity of nanoparticles to normal tissue and organs.

Furthermore, several issues concerning the obtention of nanosized carriers loading natural triterpenoids could be addressed in the future, thus improving and broadening the desired outcomes. The expansion of encapsulation of other natural triterpenes with anti-arthritic activity, the rise of compound tissue concentration via active targeting, or the use of stimuli-responsive nanocarriers for a better-controlled release, may contribute to a better response of triterpenoid compounds to the several forms of arthritis.

The present review discussed the appealing approach of encapsulating triterpenic compounds in nanocarriers regarding their anti-arthritic efficacy. Taking into account the serious health problems imposed by arthritis, which comprises a myriad of chronic diseases with high world prevalence rates, and given the promising reports in the literature on the use of nanoscale carriers to deliver triterpenoid compounds, it is our belief that it could spur interest in considering interdisciplinary research on RA, OA, and gout.

## Figures and Tables

**Table 2:** Polymeric nanoparticles employed to improve bioavailability and/or therapeutic effect of antiarthritic natural triterpenoids.

Triterpenoid/Drug Delivery System	Preparation Method	Characterization	Cell/Animal Model	Results	Ref.
Celastrol (**6**) MMP-9-responsive PLGA-RGD-PEG NPs (PRNPs)	**6**-NPs (PLGA only) and **6**-RNPs (PLGA + RGD) were prepared using the emulsion/solvent evaporation method. **6**-PRNPs (PLGA + RGD + PEG) were obtained by linking **6**-RNPs to the cleavable peptide PEG2000-MMP-9 using the water phase reaction method.	**6**-NPs (PLGA only): size 155.7 ± 4.9 nm. **6**-RNPs (PLGA + RGD): size 154.1 ± 4.6 nm, ZP −3.2 ± 0.6 mV. **6**-PRNPs (PLGA + RGD + PEG): size 162.2 ± 6.6 nm, ZP −5.3 ± 0.4 mV. EE near 90% for all NPs.	LPS-activated murine BMDMs and osteoclasts. Human synovial macrophages and osteoclasts from late-stage RA patients. Male Wistar rats (n = 3) with early-stage or advanced adjuvant-induced arthritis (AA).	**6**-PRNPs selectively targeted both osteoclasts and inflammatory macrophages through ligand (RGD)-receptor (αvβ3 integrin) interactions upon cleavage of the PEG chains by MMP-9 and exposure of the RGD peptide, resulting in high levels of apoptosis. In AA rats, **6**-PRNPs showed an arthritic joints-specific distribution, promoted inflammatory remission, relieving ankle and paw swelling, and reversed bone erosion in the inflamed joints of animals with advanced arthritis. Reduced RANKL expression and decreased RANKL/OPG ratio with significant accumulation of OCN-positive osteoblasts and increased expression of ALP in the arthritic joints was observed, along with reduced secretion of TNF and IL-1β in ankle joints. **6**-PRNPs showed reduced cellular uptake in the absence of MMP-9 with negligible off-target apoptosis and were considered safe.	[72]
Celastrol (**6**) Human serum albumin-Kolliphor^®^HS15 nanoparticles (HSA-HS15 NPs)	Addition of aqueous phase (12.5 mg Kolliphor^®^ HS15 and 20% *w*/*v* HSA in 5 mL water) to organic phase (3.5 mg **6** and 22.5 mg soybean oil in 1 mL DCM)	**6**-HSA-HS15 NPs: size 78.5 ± 2.7 nm, PDI 0.154 ± 0.02, ZP −1.45 ± 1.19 mV, DL 2.4 ± 0.97%, EE 94.6 ± 1.7%. **6**-HSA NPs: size 74.9 ± 2.7 nm, PDI 0.164 ± 0.05, ZP −21.4 ± 1.34 mV, DL 2.6 ± 0.06%, EE 94.4 ± 2.3%	Adjuvant-induced arthritis (AA) in male SD rats (n = 7)	**6**-HSA-HS15 NPs accumulated at the inflamed synovium of AA rats due to the ELVIS effect and targeting ability of albumin. At the inflammation site, **6**-HSA-HS15 NPs were phagocytosed by the activated macrophages, releasing celastrol with subsequent inhibition of pro-inflammatory cytokines (TNF-α and IL-1β). The therapeutic effect of **6**-HSA-HS15 NPs was superior when compared with free **6** and **6**-HSA NPs formulated without Kolliphor^®^HS15 addition.	[73]
Glycyrrhizin (**7**) in combination with budenoside Aminocellulose (AC)-grafted-polycaprolactone (PCL) coated gelatin NPs (PCL-AC-gel NPs)	**7**-loaded gelatin NPs prepared by nanoprecipitation method and physically coated with budenoside-loaded PCL-AC layer.	**7**-budenoside -loaded PCL-AC-gel NPs: size 210–225 nm, PDI 0.35, ZP 20.3 mV. Blank NPs: size 187.5 nm, PDI 0.25, ZP 11.2 mV. DL 13.85% (**7**) and 16.04% budenoside. EE 69.22% (**7**) and 80.20% budenoside	CIA in female Wistar rats (n = 6)	Release studies in PBS (pH 7.4) at room temperature showed that dual-loaded NPs exhibited a sustained release of both **7** and budenoside, with almost 50% of the drugs being released in 24 h. The dual drug-loaded NPs reduced inflammatory cells infiltration, joint swelling, collagen destruction and bone erosion in CIA rats, and decreased the levels of pro-inflammatory mediators (iNOS, COX-2, TNF-α and IL-1β). The nanocarriers exerted superior therapeutic efficacy compared to the free drugs without toxic effects to liver and kidney.	[74]
11-keto-β-boswellic acid (**8**) PLGA NPs	**8**-loaded PLGA NPs prepared by emulsion-diffusion-evaporation method with polymer/drug ratio of 50:10, PVA (1% *w*/*v*) as stabilizer and trehalose (10% *w*/*v*) as cryoprotectant.	Size 152.6 ± 4.4 nm; PDI 0.194 ± 0.085; ZP −3.18 ± 0.38 mV; EE 79.7 ± 1.99%.	Carrageenan-induced hind paw oedema in SD rats (n = 5).	**8**-loaded PLGA NPs showed a 7-fold increase in oral bioavailability and improved pharmacokinetic profile compared with **8** alone. The nanoformulation exhibited enhanced anti-inflammatory activity in vivo attaining 60.8% inhibition of paw volume after 5 h of oedema induction compared with 34.9% for free **8** and 43.5% for ibuprofen. The in vitro release profile of **8** in PBS (pH 7.4) at 37 °C showed an initial burst release effect attributed to surface association.	[75]
3-*O*-acetyl-11-keto-β-boswellic acid (**9**) PLGA NPs	**9**-loaded PLGA NPs prepared by emulsion-diffusion-evaporation method with polymer/drug ratio of 50:10, PVA (1% *w*/*v*) as stabilizer and trehalose (10% *w*/*v*) as cryoprotectant.	Size 179.6 ± 7.51 nm; PDI 0.276 ± 0.057; EE 82.5 ± 1.55%.	Carrageenan-induced hind paw oedema in SD rats (n = 5).	**9**-loaded PLGA NPs showed a 6-fold increase in oral bioavailability and improved pharmacokinetic profile compared with **9** alone. The nanoformulation exhibited enhanced anti-inflammatory activity in vivo attaining 67.0% inhibition of paw volume after 5 h of oedema induction compared with 40.5% for free **9** and 43.5% for ibuprofen. The in vitro release profile of **9** in PBS (pH 7.4) at 37 °C showed an initial burst release effect attributed to surface association.	[76]
Ginsenoside Rb1 (**10**) Polymeric (PCL) nanocapsules (nanoGsRb1)	Polymeric nanocapsules made by mixing organic phase containing PCL (100 mg), sorbitan stearate (400 mg), propanone (25 mL) and **10** (80 mg) with aqueous phase containing glycol (75.9 mg in 49 mL water)	Size 173.1 ± 19.7 nm; ZP 36.9 mV; EE 99.79%	MSU-induced gouty arthritis in male SD rats (n = 8)	The nanoformulation showed a slower release rate of **10** under physiological conditions (PBS pH 7.4, 37 °C) compared with the free form (15.84% vs. 32.74% in 1 h, respectively). In the MSU-induced gouty arthritis rat model, nano**10** was more efficient at decreasing NLRP3 and NF-κB expressions and at increasing IκB-α than **10** alone. Nano-**10**, and to a lesser extent free **10**, reduced the expressions of TNF-α, IL-1β, and IL-6 in the inflamed rat joint and ameliorated paw swelling and bone destruction.	[77]

**Table 3:** Polymeric micelles employed to improve bioavailability and/or therapeutic effect of antiarthritic natural triterpenoids.

Triterpenoid/ Drug Delivery System	Preparation Method	Characterization	Cell/Animal Model	Results	Ref.
Celastrol (**6**) Dextran sulphate-PVGIG-Celastrol (DPC) nanomicelles	Polymeric micelles (DPC) made by self-assembly of amphiphilic polymer comprising **6** as hydrophobic core and dextran sulfate (DS) as both hydrophilic block and SR-A ligand for targeting activated macrophages, linked by the MMP-2-responsive peptide PVGLIG. **6**-loaded DPC micelles (DPC@**6**) prepared by the dialysis method.	DPC nanomicelles: CMC 0.1762 mg/mL. DPC@**6** nanomicelles: size 189.9 nm, DPI 0.092, ZP −11.91 mV. DL 3.46% EE 38.07%	LPS-activated RAW264.7 cells and RA-FLSs. AA in SD rats (n = 5).	In vitro release studies showed that in the presence of MMP-2 enzyme, DPC@**6** nanomicelles delivered 78% of 6 after 72 h but only 30% in the absence of the enzyme. DPC@**6** increased the accumulation of **6** in RAW264.7 cells, improved **6** release rate and promoted apoptosis of RA-FLSs in a concentration-dependent manner. DPC@**6** reduced the swelling rate, synovial inflammation, and bone erosion in AA rat joints more effectively than free **6** with negligible systemic toxicity.	[78]
Celastrol (**6**) ROS-responsive PEGylated bilirubin nanoparticles (BRNPs)	BRNPs prepared by self-assembly of amphiphilic polymer obtained by conjugating antioxidant and hydrophobic bilirubin (BR) with hydrophilic mPEG. **6**-loaded BRNPs (**6**/BRNPs) prepared by self-assembly upon mixing PEGylated BR (25 mg/mL) with **6** (10 mg/mL) in DMSO/aqueous solution.	BRNPs: CMC 7 µg/mL. **6**/BRNPs: size 68.6 nm, PDI 0.155) ZP −7.3 mV. DL 6.6% EE 72.6%	LPS-activated macrophages (RAW264.7 cells). AA in male SD rats (n = 5).	In vitro release studies showed faster and sustained-release in the presence of H_2_O_2_, with up to 60% release over the first 4 h in 100 mM H_2_O_2_. BRNPs were efficiently internalized by activated RAW264.7 cells involving clathrin- and caveolin-dependent endocytic pathways. **6**/BRNPs effectively scavenged intracellular ROS and down-regulated NO level after internalization by activated macrophages. **6**/BRNPs ameliorated hind paw oedema in AA rats, inhibited TNF-α and IL-1β serum levels, and prevented cartilage erosion with higher efficacy compared to free **6**. Ex-vivo studies in AA rats revealed that BRNPs showed enhanced accumulation in the arthritic joints and lessen the distribution in liver, spleen and lung when compared to free **6**.	[79]
Celastrol (**6**) Polymeric micelles	ROS-responsive PEG-*b*-PPS (PEPS) copolymer synthesized via multistep reaction. **6**-loaded micelles (**6**-PEPS) prepared by self-assembly upon addition of **6** (5 mg) to PEPS (50 mg) in water	Mean size 135 nm, PDI 0.29, DL 4.71%	LPS-stimulated macrophages (RAW246.7 cells). CIA in male DBA/1 mice (n = 5–8).	**6**-PEPS down-regulated the transcription of iNOS, TNF-α and IL-6 genes in LPS-activated macrophages, inhibiting NF-κB and Notch1 pathways and preventing M1 activation. Intravenous administration of **6**-PEPS to CIA mice led to NP accumulation in the inflamed joints, reducing synovial inflammation, bone erosion and cartilage destruction, ameliorating arthritis symptoms. **6**-PEPS treatment also decreased mice serum levels of pro-inflammatory cytokines TNF-α, IL-1β and IL-6 while up-regulating anti-inflammatory cytokines TGF-β1 and M-CSF. Dissection of major organs (heart, liver, spleen, lung, and kidney) at day 51 revealed normal physiological structure and cell morphology, demonstrating that **6**-PEPS were not cytotoxic.	[80]
3-*O*-acetyl-11-keto-β-boswellic acid (**9**) Polymeric nanomicelles	Radical polymerization using N-isopropylacrylamide (NIPAAM), vinylpyrrolidone and acrylic acid at 65:30:5 molar ratio and methylene bis-acrylamide as cross-linker; transdermal gel of **9** (20 g)-loaded nanomicelles prepared using 1% *w*/*v* Carbopol 940.	Size 45 nm; DL 45%; EE 90%	Excised abdominal rat skin; carrageenan-induced hind paw oedema in Wistar rats (n = 5); AA in Wistar rats (n = 5).	In vitro sustained release of **9** from nanomicelles at physiological pH 7.4. Increased rat skin permeability in vitro. Enhanced anti-inflammatory activity in vivo (81.1% oedema inhibition at 24 h compared with 44.1% for plain **9** gel). Enhanced anti-arthritic activity in vivo (60.6% inhibition of paw volume increase at day 13 compared with 33.3% for the plain **9** gel). Enhanced therapeutic effects attributed to small size and mucoadhesiveness of nanomicelles, enhanced skin permeation and sustained release.	[81]

## Data Availability

Data sharing is not applicable.

## Supplementary Materials

The following supporting information can be downloaded at: https://www.mdpi.com/article/10.3390/ph17010054/s1, Table S1. Global prevalence, incidence and years lived with disability (YLDs) attributable to arthritis between 2010 and 2019; Table S2. Characteristics of some common types of arthritis addressed in this review; Table S3. Main animal models of experimentally induced arthritis; Table S4. Physicochemical properties of major antiarthritic triterpenes covered in the present review. Data from PubChem (National Institutes of Health) or CAS DataBase List (Chemical Book) unless otherwise stated; Table S5. Pharmacokinetic parameters for nanoformulations of anti-arthritic triterpenes in preclinical studies obtained from plasma or joint fluid (values in brackets) samples. References [4,73,75,76,87,97,98,99,104,107,112,113,114,115,116,117] are cited in the supplementary materials.

## Abbreviations

AA, adjuvant-induced arthritis; AC, aminocellulose; ALP, alkaline phosphatase; BMDM, bone marrow-derived macrophage; BR, bilirubin; CII, collagen type 2; CFA, complete Freund’s adjuvant; Chol, cholesterol; CIA, collagen-induced arthritis; CMC, critical micelle concentration; COX, cyclooxygenase; CPP, cell-penetrating peptide; CTAB, cethyltrimethylammonium bromide; DCM, dichloromethane; DL, drug loading; DMSO, dimethyl sulfoxide; DOTAP, 1,2-dioleoyl-3-trimethylammonium propane; DS, dextran sulphate; DSPE, distearoylphosphatidyl ethanolamine; EE, encapsulation efficiency; EPC, egg phosphatidylcholine; FA, folic acid; FLS, fibroblast-like synoviocyte; GMS, glycerin monostearate; GPTMS, 3-glycidoxypropyltrimethoxysilane; GSH, glutathione; HA, hyaluronic acid; HMSN, hollow mesoporous silica nanoparticle; HPMC, hydroxypropylmethyl cellulose; HSA, human serum albumin; IL, interleukin; iNOS, inducible nitric oxide synthase; i.p., intraperitoneal; i.v., intravenous; LPS, lipopolysaccharide; M-CSF, macrophage colony-stimulating factor; MIA, monoiodoacetate; MMP, matrix metalloproteinase; mPEG, poly(ethylene glycol) monomethyl ether; MSN, mesoporous sílica nanoparticle; MSU, monosodium urate; NaCMC, sodium carboxymethyl cellulose; NF-kB, nuclear factor kappa-light-chain-enhancer of activated B cells; NIPAAM, N-isopropylacrylamide; NLC, nanostructured lipid carrier; NLRP3, nucleotide-binding domain, leucine-rich repeat and pyridine domain-containing 3; NO, nitric oxide; Notch1, neurogenic locus notch homolog protein 1; NP, nanoparticle; OA, osteoarthritis; OCN, osteocalcin; OPG, osteoprotegerin; PBS, phosphate buffered saline; PC, phosphatidylcholine; PCL, poly(ε-caprolactone); PDI, poly dispersity index; PEG, poly(ethylene glycol); PK, pharmacokinetic; PLGA, poly(D,L-lactic-co-glycolic acid); p.o., per os (by mouth); PPS, poly(propylene sulphide); PVA, poly(vinyl alcohol); RA, rheumatoid arthritis; RANKL, receptor activator of nuclear factor kappa-B ligand; ROS, reactive oxygen species; s.c., subcutaneous; SD, Sprague-Dawley; SDC, sodium deoxycholate; SLN, solid lipid nanoparticle; SLT, soybean lecithin; SR-A, scavenger receptor class A; TEA, triethanolamine; TEOS, tetraethyl orthosilicate; TGF, transforming growth factor; TNF, tumour necrosis factor; TPGS, D-α-tocopheryl polyethylene glycol succinate; ZP, zeta potential.
