# Supplementary material for: Triterpenes Drug Delivery Systems, a Modern Approach for Arthritis Targeted Therapy
Source: Pharmaceuticals (Basel). 2023 Dec 28;17(1):54. doi: 10.3390/ph17010054 (PMC10819636; doi:10.3390/ph17010054)
Supplement: Supplementary file 1 [file pharmaceuticals-17-00054-s001.zip › pharmaceuticals-2712951-supplementary.pdf]

Triterpenes drug delivery systems, a modern approach for arthritis targeted therapy

Célia Faustino[1,#], Noélia Duarte [1,*], and Lídia Pinheiro [1,*]

[1] iMed.ULisboa, Research Institute for Medicines, Faculdade de Farmácia, Universidade de Lisboa, Avenida Prof. Gama Pinto, 1649-003 Lisbon, Portugal. C.F. cfaustino@ff.ulisboa.pt; N.D. mduarte@ff.ulisboa.pt; L.P. lpinheiro@ff.ulisboa.pt

* Correspondence: N.D. mduarte@ff.ulisboa.pt; L.P. lpinheiro@ff.ulisboa.pt

Supplementary Materials

Table S1. Global prevalence, incidence and years lived with disability (YLDs) attributable to arthritis between 2010 and 2019. Data from Global Burden of Disease Collaborative Network, 2020

Table S2. Characteristics of some common types of arthritis addressed in this review.

Table S3. Main animal models of experimentally induced arthritis.

Table S4. Physicochemical properties of major antiarthritic triterpenes covered in the present review. Data from PubChem (National Institutes of Health) or CAS DataBase List (Chemical Book) unless otherwise stated.

Table S5. Pharmacokinetic parameters for nanoformulations of anti-arthritic triterpenes in preclinical studies obtained from plasma or joint fluid (values in brackets) samples.

Table S1. Global prevalence, incidence and years lived with disability (YLDs) attributable to arthritis between 2010 and 2019. Data from Global Burden of Disease Collaborative Network, 2020 [1].

**Type of arthritis**	**Gender**	**Prevalence**<br>**Cases** (millions)	**Incidence**<br>**Cases** (millions)	**YLDs**<br>Counts (millions)
Osteoarthritis	Male	210 (30.4%)	17.1 (25.1%)	7.39 (30.9%)
	Female	317 (25.6%)	24.4 (20.9%)	11.6 (25.4%)
	Overall	528 (27.5%)	41.5 (22.6%)	18.9 (27.5%)
Rheumatoid arthritis	Male	5.39 (23.9%)	0.330 (20.1%)	0.716 (23.3%)
	Female	13.2 (21.7%)	0.744 (17.3%)	1.72 (21.2%)
	Overall	18.6 (22.3%)	1.07 (18.1%)	2.43 (21.8%)
Gout	Male	40.7 (32.1%)	6.88 (30.2%)	1.27 (31.3%)
	Female	13.2 (29.8%)	2.34 (25.8%)	0.402 (29.1%)
	Overall	53.9 (31.5%)	9.22 (29.1%)	1.67 (30.7%)

Table S2. Characteristics of some common types of arthritis addressed in this review.

Arthritis type	Osteoarthritis	Rheumatoid arthritis	Gout
Commonly affected joints	Knee, hand (small joints of the fingers and the base of the thumb), hip, neck, lower back and big toe.	Hands, wrists, knees and feet, typically in symmetrical pattern.	Big toe (most often), lesser toe, ankle and knee, usually in only one joint at a time.
Symptoms	Pain (during activity, after long activity or at the end of the day), early morning stiffness (lasting less than 30 min), swelling, crepitus and decreased range of motion that may improve after movement.	Pain, tenderness, early morning stiffness, and swelling involving multiple (peripheral) joints bilaterally, low-grade fever, fatigue and weight loss.	Severe pain (often at night) of rapid onset (6–12 h), swelling, redness and warmth at the affected joint.
Main risk factors	Joint injury/overuse, advancing age, female sex, overweight/obesity, positive family history/genetics, race/ethnicity, bone mineral density and lack of physical activity.	Advancing age, female sex, positive family history/genetics, overweight/obesity, smoking, particulate matter exposure, infectious agents, microbiome dysbiosis, stress and pro-inflammatory diet (rich in fried foods, processed foods, refined carbohydrates, sodas and red meat).	Hyperuricemia, genetics, advancing age, male sex, race/ethnicity, overweight/obesity, purine-rich diet (red meat, organ meat and some kinds of seafood), alcohol intake, high-fructose/sugar sweetened beverages, certain drugs (diuretics, low-dose aspirin and immunosuppressants), infections and gut

			microbiome dysbiosis.
Common comorbidities	Hypertension, cardiovascular disease, obesity, dyslipidaemia, type 2 diabetes and depression.	Cardiovascular disease, lymphoma, interstitial lung disease, pulmonary fibrosis, vasculitis, metabolic syndrome, type 2 diabetes, atherosclerosis, osteoporosis, anaemia, dry keratoconjunctivitis and depression.	Cardiovascular disease, hypertension, type 2 diabetes, hyperlipidaemia, obesity, metabolic syndrome and chronic kidney disease.
Pharmacotherapy	NSAIDs (e.g., aspirin, ibuprofen, naproxen, ketorolac, diclofenac and celecoxib); paracetamol; topical capsaicine; Duloxetine (antidepressant); corticosteroid injections; Intra-articular injections of hyaluronic acid	Disease-modifying anti-rheumatic drugs (DMARDs); NSAIDs; glucocorticoids	NSAIDs (non-selective or COX-2 selective); glucocorticoids; colchicine;<br>XOD inhibitors (e.g., allopurinol and febuxostat)
Other treatments/recommended lifestyle	Regular aerobic exercise, weight loss, healthy and non-inflammatory diet physical therapy, hot or cold therapies, acupuncture, osteopathic manipulation, chiropractic care and massage; glucosamine and chondroitin sulphate; Surgical intervention	Healthy lifestyle, regular exercise, non-smoking, reduced stress and an anti-inflammatory diet	Topical ice;<br>weight loss and dietary restrictions, avoiding fat, purine-rich proteins, alcohol consumption and sugar/fructose-sweetened drinks

Table S3. Main animal models of experimentally induced arthritis.

Arthritis type	Model	Species	Method	Characteristics
Osteoarthritis	*Surgically-induced models*			
	Anterior cruciate ligament transection (ACLT)	Dogs	Transection of the anterior cruciate ligament (ACL)	Causes joint destabilization leading to post-traumatic OA.<br>Mimics the degradation of articular cartilage after ACL rupture.<br>OA lesions develop more slowly compared with meniscectomy model.
	Destabilization of the medial meniscus (DMM)	Rats, mice, rabbits	Transection of the medial meniscotibial ligament	Lesions progress from mild-to-moderate OA at 4 weeks to moderate-to-severe OA at 8 weeks post-surgery.
	Medial meniscal tear (MMT)	Rats, guinea pigs	Transection of the medial collateral ligament in the knee	Causes proteoglycan and chondrocyte loss leading to cartilage degradation.<br>Progressive degeneration with tibial cartilage degradation after 3–6 weeks.
	Partial medial meniscectomy (MMx)	Guinea pigs, rabbits, dogs	Partial meniscectomy of the knee medial meniscus (or lateral meniscus in rabbits)	Cause destabilization of the joint leading to rapid degeneration and more severe OA compared with the ACLT model.<br>Rabbits preferentially load the lateral meniscus, developing more severe OA upon lateral meniscectomy.

			Total meniscectomy (dogs) leads to more severe OA than partial meniscectomy.
Ovariectomy	Mice, rats, guinea pigs, rabbits, sheep	Bilateral ovariectomy	Induces oestrogen deficiency mimicking post-menopausal phenotype.<br>Post-menopausal females are known to develop osteoporosis leading to OA.
Chemically-induced models			
Monosodium iodoacetate (MIA)-induced arthritis	Mice, rats	Intra-articular MIA injection	Inhibition of the glycolytic enzyme glyceraldehyde-3-phosphate dehydrogenase in chondrocytes.<br>Causes chondrocyte death, osteophyte formation, articular cartilage degradation, rapid inflammation and hyperalgesia lasting for 7 days, followed by chronic musculoskeletal pain at the 10th day post-injection.
Collagenase-induced arthritis	Mice, rats, rabbits	Intra-articular collagenase injection	Breaks down type I collagen within the cartilage matrix of tendons and ligaments, leading to loss of structural integrity, joint instability, inflammation, and pain.
Kaolin-Carrageenan (K/C)-induced arthritis	Rats, mice, guinea pigs, cats, macaques	Intra-articular injections of kaolin and carrageenan on one knee joint	Acute onset aseptic inflammatory monoarthritis with degeneration of hyaline articular cartilage, synovial inflammation and pain, resembling OA.<br>It develops rapidly (within hours) and persists for weeks.<br>A modification of the K/C arthritis model is the knee joint monoarthritis induced by intra-articular injection of carrageenan alone. The time course of the carrageenan-induced arthritis is shorter (hours to days) and the cartilage

				damage less pronounced than in the K/C model.
	Quinolone-induced arthritis	Guinea pigs	Oral or parenteral administration of quinolone antibiotics	Oral quinolone antibiotics (which target bacterial DNA gyrase) are known to damage epiphyseal growth plate of immature animals.<br>Systemic administration causes loss of cartilage proteoglycans and chondrocytes.
Rheumatoid Arthritis	Adjuvant-induced arthritis (AA)	Rats	Subcutaneous or intradermal immunization (tail root or footpad) with complete Freund's adjuvant (CFA) containing high concentration (10–20 mg/mL) of heat-killed *Mycobacterium tuberculosis* $H_{37}R_a$ (Mtb) emulsified in mineral oil.	Activates T-cell response to Mtb heat-shock protein 65 (Bhsp65).<br>Induces chronic polyarthritis with synovitis, angiogenesis, pannus formation, cartilage and bone erosion, bone matrix resorption and joint ankylosis. Granuloma formation in periarticular tissue. Anorexia and weight loss.<br>Biphasic time course, initial acute inflammation (within hours) subsiding after 3–5 days followed by chronic systemic inflammation exhibiting a relapsing-remitting course after the initial 2 weeks that can persist for several months.<br>A less severe CFA mono-arthritis model induced by intra-articular (knee) injection of CFA was developed to avoid the systemic disease and chronic pain of the classical polyarthritis model. Chronic localised monoarthritis develops 10–14 days after immunization, with synovitis, pannus formation, cartilage damage and bone erosion, which persists for several weeks.
	Collagen-induced	Rats, mice,	Intradermal or	T cell and B cell-specific response to CII. Breach of

arthritis (CIA)	macaques	subcutaneous immunization (tail root) with heterologous type II collagen (CII) emulsified in CFA, usually followed by a booster injection of CII emulsified in IFA, after 14 or 21 days, to ensure high CIA incidence.	tolerance against self-collagen with production of autoantibodies, including ACPA and R_F, resembling human RA.<br><br>Acute and self-remitting erosive polyarthritis 21–28 days post-immunization with proliferative synovitis, infiltration of polymorphonuclear and mononuclear cells, pannus formation, and severe erosions of cartilage and bone.<br><br>Susceptibility to CIA in rodent strains is dependent on MHC class II haplotype.
Collagen antibody-induced arthritis (CAIA)	Mice	Intravenous (tail vein) or intraperitoneal administration of a cocktail of anti-CII monoclonal antibodies, followed by intraperitoneal LPS injection (booster) after 3 days.	Self-limiting polyarthritis with synovial hyperplasia, macrophage and polymorphonuclear cell infiltration, pannus formation, and cartilage and bone erosion.<br><br>Not associated with T cell and B cell response, passive transfer of anti-CII mAbs bypasses the adaptive immune system and disease is mainly driven by complement activation.<br><br>Rapid disease onset (within 48 h) with nearly 100% incidence, reaching maximum severity in 5–7 days with symptoms persisting for 2 weeks.<br><br>Not restricted to the MHC class haplotype, higher synchronicity and shorter timeframe compared with the CIA model.<br><br>Intraperitoneal injection results in a slower disease onset

			and less severe arthritis, with a shorter period of active inflammation, compared with intravenous administration.
Antigen-induced arthritis (AIA)	Rats, mice	Subcutaneous immunization with antigen (e.g., methylated bovine serum albumin) emulsified in CFA with subsequent challenge by intra-articular (knee) injection of the same antigen in saline after 21 days.	Unilateral T cell-dependent monoarthritis model, with disease onset 1–3 days after intra-articular injection, which induces an acute inflammatory reaction by development of antigen-specific $CD4^+$ T cells, leading to chronic arthritis with synovial hyperplasia, infiltration of mononuclear cells, cartilage destruction and bone erosion.<br><br>Cationic mBSA can be retained by the negatively charged hyaline cartilage, which acts as a reservoir, leading to a delayed release of antigens that promotes *in situ* immune complex formation and deposition at the cartilage surface.<br><br>T cell-mediated flares can be induced by local or systemic rechallenge with low-dose antigen, exhibiting a severe erosive phenotype.
Pristane-induced arthritis (PIA)	Rats, mice	Single intraperitoneal or intradermal (tail root) injection of pristane	T cell-dependent chronic polyarthritis in peripheral joints with synovial infiltration of mononuclear and polymorphonuclear cells, pannus formation, cartilage and bone erosion, and ankylosis of the joints.<br><br>Disease onset after 1–2 weeks after pristane (non-immunogenic adjuvant) injection.
Proteoglycan-induced arthritis (PGIA)	Rats, mice	Intraperitoneal immunization with human (arthritic) cartilage-derived proteoglycans (PGs),	$CD4^+$ T cell and B cell-specific response to PGs, antibodies appear 2 weeks after immunization and peak at 5–6 weeks. R_F positive.<br><br>Progressive chronic polyarthritis with symmetrical

		emulsified in adjuvant (CFA or DDA).	synovitis, synovial mononuclear cell and neutrophil infiltration, pannus formation, and deposition of immune complexes in the joints with persistent joint inflammation, cartilage and bone erosion, and development of ankylosing spondylitis.<br><br>Use of the synthetic adjuvant DDA results in earlier disease onset and increased disease severity, without granuloma formation and tissue irritation compared with immunogenic CFA.
Streptococcal cell wall-induced arthritis (SCWA)	Rats, mice	Intra-articular injection(s) of streptococcal cell wall (SCW) fragments	Single intra-articular injection leads to acute inflammation with flares obtained by subsequent intravenous injection of SCW fragments while multiple intra-articular injections lead to chronic monoarthritis.<br><br>Joint inflammation progresses from a T cell-independent acute phase (1–5 days) through a remission phase (day 10) followed by a spontaneous T cell-dependent reactivation phase (day 14) and chronic phase at 4 weeks, which persists for months.<br><br>Rapid onset (1 day), development of anti-SCW antibodies.<br><br>Polyarthritis can be induced by intraperitoneal injection of SCW fragments in rats.
Zymosan-induced arthritis (ZIA)	Mice, rats	Intra-articular injection of zymosan	Zymosan, a polysaccharide from the cell wall of *Saccharomyces cerevisae*, binds to TLR2 in macrophages, leading to induction of pro-inflammatory cytokines, arachidonate mobilization and activation of complement

				via the alternative pathway.<br>Causes proliferative inflammatory monoarthritis with mononuclear cell infiltration, synovial hypertrophy, and pannus formation.<br>Disease peaks at day 3 and subsides by day 7, relapsing after day 25.
Gouty arthritis	Potassium oxonate-induced hyperuricemia	Mice, rats	Intraperitoneal injection of potassium oxonate (250–300 mg/kg), once daily, for 7 days.	Uricase inhibition by potassium oxonate leads to increased serum urate levels.<br>Association with hypoxanthine and/or purine-rich diet (yeast extract) further increments uric acid concentrations in serum.
	Air pouch gout model	Mice, rats	Formation of an air pouch in the mouse back by subcutaneous injection of sterile air (5 mL on day 0 and 3 mL on day 3 to keep the pouch inflated) followed by direct injection of MSU crystals into the air pouch on day 7.	Mechanical disruption of the subcutaneous connective tissue by repeated air injections creates a bursal space (air pouch) within 6 days that resembles the human synovium.<br>A suspension of MSU crystals in PBS injected into the air pouch induces NLRP3 inflammasome activation, neutrophil recruitment, and IL-1β secretion.
	Footpad gout model	Mice, rats	Subcutaneous injection of MSU crystals on plantar surface of hind foot	Acute inflammation with paw oedema and tenosynovitis.
	MSU crystal-	Mice, rats	Intraperitoneal injection of	Acute peritoneal inflammation (peritonitis) with neutrophil

	induced peritonitis		MSU crystals	infiltration and IL-1β secretion.
	MSU crystal-induced arthritis	Mice, rats, rabbits	Intra-articular injection of MSU crystals in one joint (ankle or knee)	Acute inflammation (synovitis) and joint swelling within 2–3 h that resolves after 3–7 days.
	Acute gout with hyperuricemia	Mice, rats	Intra-articular injection of MSU crystals after long-term treatment (24–28 days) with potassium oxonate by oral gavage.	Inhibition of uricase by potassium oxonate combined with MSU crystal-induced NLRP3 inflammasome activation leads to increased serum uric acid levels, pro-inflammatory cytokines release, synovial inflammation, hyperplasia, joint swelling and cartilage degeneration.

ACPA, anti-citrullinated peptide antibodies; DDA, dimethyldioctadecyltrimethylammonium bromide; IFA, incomplete Freund's adjuvant; LPS, lipopolysaccharide; mBSA, methylated bovine serum albumin; MHC, major histocompatibility complex; R_F, rheumatoid factor.

Table S4. Physicochemical properties of major antiarthritic triterpenes covered in the present review. Data from PubChem (National Institutes of Health) or CAS DataBase List (Chemical Book) unless otherwise stated.

Triterpene	MW (g/mol)	m.p. (° C)	pK_a (predicted)	Water solubility, 25 °C (µg/mL)	logP (predicted)
Asiatic acid	488.7	325–330	4.66	59.8 [2]	5.7
Celastrol	450.6	219–230	4.78	13.25 ± 0.83 at 37 °C [3]	5.9
β-boswellic acid	456.7	130–135	4.50	0.49 (estimated)	8.3
11-keto-β-boswellic acid	470.7	190–191	4.46	1.5 (estimated)	7.2
3-*O*-acetyl-11-keto-β-boswellic acid	512.7	271	4.28	0.45 (estimated)	7.2
Ginsenoside Rb1	1109.3	197.5	12.85	15.32 ± 0.91 mg/mL at 20 °C [4]	0.3
Ginsenoside C-K	622.9	> 149 (dec)	-	35.2 ± 4.3 at 37 °C [5]	5.6
Glycyrrhizin	822.9	220 (dec)	2.76	54.5 (estimated)	3.7

Oleanolic acid	456.7	310	5.11) [6]	1.748 µg/L [7]	6.32 [6]
Ursolic acid	456.7	292 (dec)	5.29 [6]	5.64 [8]	6.46 [6]

Table S5. Pharmacokinetic parameters for nanoformulations of anti-arthritic triterpenes in preclinical studies obtained from plasma or joint fluid (values in brackets) samples.

Triterpene	Formulation	Species (population)	Adm. route	Dose (mg/kg)	T_{max} (h)	C_{max} (μg/mL)	$AUC_{0\to\infty}$ (μg·h/mL)	$t_{1/2}$ (h)	MRT (h)	*F* (%)	Ref.
11-keto-β-boswellic acid (**8**)	PLGA NPs	SD rats (n = 5)	Oral	50	3.0	15.40 ± 0.61	90.63 ± 2.37	6.20 ± 0.04	6.93 ± 0.25	705.84	[9]
	Suspension				1.50	3.48 ± 0.12	12.84 ± 0.53	2.48 ± 0.02	3.72 ± 0.50	-	
3-*O*-acetyl-11-keto-β-boswellic acid (**9**)	PLGA NPs	SD rats (n = 5)	Oral	50	2.00	3.13 ± 0.13	34.44 ± 0.84	10.13 ± 0.09	NA	914.37	[10]
	Suspension				1.50	0.585 ± 0.024	3.77 ± 0.01	4.76 ± 0.05	NA	-	
Celastrol (**6**)	HSA-HS15 NPs	Male SD rats (n = 5)	Intravenous	2	0.083	36.36 ± 0.86	90.17	5.63	NA	361.00	[11]
	HSA NPs				0.083	26.15 ± 0.54	49.82	4.77	NA	199.45	
	Solution				0.083	11.78 ± 0.46	24.98	2.20	NA	-	
Celastrol (**6**)	HA-coated bilosomes	Arthritic SD rats (n = 6)	Intravenous	10	NA	25.24 ± 0.57 (4.854 ±	112.19 ± 0.85 (11.783 ±	5.78 ± 0.38	8.075 ± 0.319	312.86 (799.9)	[12]

						0.674)	0.388)				
	Uncoated bilosomes				NA	23.57 ± 0.49 (2.671 ± 0.367)	76.19 ± 1.30 (7.075 ± 0.213)	5.98 ± 0.47	7.922 ± 0.681	212.47 (480.3)	
	Solution				NA	22.62 ± 0.71 (0.652 ± 0.146)	35.86 ± 0.53 (1.473 ± 0.402)	6.15 ± 0.22	7.073 ± 0.767	-	
Celastrol (**6**)	SMEDDS	Male SD rats (n = 6)	Oral	4	1.08 ± 0.061	0.98 ± 0.072	3.12 ± 0.07	0.52 ± 0.065	3.13 ± 0.081	567.27	[3]
	SMEDDS dispersible tablets				1.16 ± 0.039	0.89 ± 0.07	3.07 ± 0.07	0.54 ± 0.054	3.68 ± 0.091	558.18	
	Suspension				0.22	0.17 ± 0.076	0.55 ± 0.12	0.52 ± 0.12	3.72 ± 0.13	-	
Oleanolic acid (**13**)	SMEDDS	Male SD rats (n = 5)	Oral	50	2.00 ± 1.00	0.210 ± 0.047	1.775 ± 0.158	NA	NA	507.03	[13]
	Tablet				2.75 ± 0.50	0.0786 ± 0.0168	0.350 ± 0.074	NA	NA	-	
Oleanolic acid (**13**)	SNEDDS	Male SD rats (n = 5)	Intragastric	50	1.50 ± 1.22	0.0998 ± 0.0426	0.601 ± 0.048	NA	4.05 ± 1.75	240.68	

											[14]
	Solution				0.80 ± 0.45	0.105 ± 0.062	0.249 ± 0.182	NA	2.30 ± 0.72	-	
Asiatic acid tromethamine salt (**14**)	SLNs	Male SD rats (n = 6)	Oral	15	0.25 ± 0.0	0.680 ± 0.234	2.347 ± 0.102	NA	5.9 ± 0.5	252.40	[15]
	Suspension				0.25 ± 0.0	0.184 ± 0.071	0.930 ± 0.238	NA	4.6 ± 0.6	-	
Celastrol (**6**)	CPP-coated NLC	Beagle dogs (n = 5)	Oral	15	1.67 + 0.58	0.483 + 0.015	2.568 + 0.343	3.09 + 0.54	4.08 + 0.37	410.39	[16]
	Uncoated NLCs				2.33 + 0.58	0.305 + 0.035	1.792 + 0.057	3.85 + 0.94	4.37 + 0.33	286.37	
	Suspension				0.96 + 0.10	0.224 + 0.032	0.626 + 0.067	1.60 + 0.13	1.65 + 0.13	-	

C_{max}, maximum plasma concentration; T_{max}, time to reach maximum plasma concentration; $AUC_{0\rightarrow\infty}$, area under the plasma concentration-time curve from zero to infinity time; $t_{1/2}$, elimination half-life; MRT, mean residence time; *F*, relative bioavailability; NA, not available; SD, Sprague-Dowley.

Note: F (%) = $[(AUC_t \times D_r)/(AUC_r \times D_t)] \times 100$, AUC_t and AUC_r are the area under the curve for test sample and reference, respectively and D_t and D_r are the doses of test sample and reference, respectively.

References

1. Institute for Health Metrics and Evaluation Global Burden of Disease Study 2019 (GBD 2019) Disease and Injury Burden 1990–2019.
2. Win, Y.Y.; Charoenkanburkang, P.; Limprasutr, V.; Rodsiri, R.; Pan, Y.; Buranasudja, V.; Luckanagul, J.A. In vivo biocompatible self-assembled nanogel based on hyaluronic acid for aqueous solubility and stability enhancement of asiatic acid. *Polymers (Basel).* **2021**, *13*, 1–18, doi:10.3390/polym13234071.
3. Qi, X.; Qin, J.; Ma, N.; Chou, X.; Wu, Z. Solid self-microemulsifying dispersible tablets of celastrol: Formulation development, charaterization and bioavailability evaluation. *Int. J. Pharm.* **2014**, *472*, 40–47, doi:10.1016/j.ijpharm.2014.06.019.
4. Wang, L.; Lu, A.P.; Yu, Z.L.; Wong, R.N.S.; Bian, Z.X.; Kwok, H.H.; Yue, P.Y.K.; Zhou, L.M.; Chen, H.; Xu, M.; et al. The melanogenesis-inhibitory effect and the percutaneous formulation of ginsenoside Rb1. *AAPS PharmSciTech* **2014**, *15*, 1252–1262, doi:10.1208/s12249-014-0138-3.
5. Zhang, Y.; Tong, D.; Che, D.; Pei, B.; Xia, X.; Yuan, G.; Jin, X. Ascorbyl palmitate/D-α-tocopheryl polyethylene glycol 1000 succinate monoester mixed micelles for prolonged circulation and targeted delivery of compound K for antilung cancer therapy in vitro and in vivo. *Int. J. Nanomedicine* **2017**, *12*, 605–614, doi:10.2147/IJN.S119226.
6. Claude, B.; Morin, P.; Lafosse, M.; Andre, P. Evaluation of apparent formation constants of pentacyclic triterpene acids complexes with derivatized β- and γ-cyclodextrins by reversed phase liquid chromatography. *J. Chromatogr. A* **2004**, *1049*, 37–42, doi:10.1016/j.chroma.2004.06.133.
7. Castellano, J.; Ramos-Romero, S.; Perona, J. Oleanolic Acid: Extraction, Characterization and Biological Activity. *Nutrients* **2022**, *14*, 623, doi:10.3390/ nu14030623 Academic.
8. Song, S.; Gao, K.; Niu, R.; Yi, W.; Zhang, J.; Gao, C.; Yang, B.; Liao, X. Binding behavior, water solubility and in vitro cytotoxicity of inclusion complexes between ursolic acid and amino-appended β-cyclodextrins. *J. Mol. Liq.* **2019**, *296*, 111993, doi:10.1016/j.molliq.2019.111993.
9. Bairwa, K.; Jachak, S.M. Nanoparticle formulation of 11-keto-β-boswellic acid (KBA): anti-inflammatory activity and in vivo pharmacokinetics. *Pharm. Biol.* **2016**, *54*, 2909–2916, doi:10.1080/13880209.2016.1194437.
10. Bairwa, K.; Jachak, S.M. Development and optimisation of 3-Acetyl-11-keto-β-boswellic acid loaded poly-lactic-co-glycolic acid-nanoparticles with enhanced oral bioavailability and in-vivo anti-inflammatory activity in rats. *J. Pharm. Pharmacol.* **2015**, *67*, 1188–1197, doi:10.1111/jphp.12420.
11. Gong, T.; Zhang, P.; Deng, C.; Xiao, Y.; Gong, T.; Zhang, Z. An effective and safe treatment strategy for rheumatoid arthritis based on human serum

albumin and Kolliphor® HS 15. *Nanomedicine* **2019**, *14*, 2169–2187, doi:10.2217/nnm-2019-0110.

12. Yang, H.; Liu, Z.; Song, Y.; Hu, C. Hyaluronic acid-functionalized bilosomes for targeted delivery of tripterine to inflamed area with enhancive therapy on arthritis. *Drug Deliv.* **2019**, *26*, 820–830, doi:10.1080/10717544.2019.1636423.

13. Yang, R.; Huang, X.; Dou, J.; Zhai, G.; Lequn, S. Self-microemulsifying drug delivery system for improved oral bioavailability of oleanolic acid: Design and evaluation. *Int. J. Nanomedicine* **2013**, *8*, 2917–2926, doi:10.2147/IJN.S47510.

14. Xi, J.; Chang, Q.; Chan, C.K.; Meng, Z.Y.; Wang, G.N.; Sun, J.B.; Wang, Y.T.; Tong, H.H.Y.; Zheng, Y. Formulation development and bioavailability evaluation of a self-nanoemulsified drug delivery system of oleanolic acid. *AAPS PharmSciTech* **2009**, *10*, 172–182, doi:10.1208/s12249-009-9190-9.

15. Lingling, G.; Yuan, Z.; Weigen, L. Preparation, optimization, characterization and in vivo pharmacokinetic study of asiatic acid tromethamine salt-loaded solid lipid nanoparticles. *Drug Dev. Ind. Pharm.* **2016**, *42*, 1325–1333, doi:10.3109/03639045.2015.1135934.

16. Chen, Y.; Yuan, L.; Zhou, L.; Zhang, Z.H.; Cao, W.; Wu, Q. Effect of cell-penetrating peptide-coated nanostructured lipid carriers on the oral absorption of tripterine. *Int. J. Nanomedicine* **2012**, *7*, 4581–4591, doi:10.2147/IJN.S34991.
